# Cultivated Olive Diversification at Local and Regional Scales: Evidence From the Genetic Characterization of French Genetic Resources

**DOI:** 10.3389/fpls.2019.01593

**Published:** 2019-12-24

**Authors:** Bouchaib Khadari, Ahmed El Bakkali, Laila Essalouh, Christine Tollon, Christian Pinatel, Guillaume Besnard

**Affiliations:** ^1^AGAP, University Montpellier, CIRAD, INRA, Montpellier SupAgro, Montpellier, France; ^2^Conservatoire Botanique National Méditerranéen de Porquerolles (CBNMed), UMR AGAP, Montpellier, France; ^3^INRA, UR Amélioration des Plantes et Conservation des Ressources Phytogénétiques, Meknès, Morocco; ^4^Établissement Public Local d’Enseignement et de Formation Professionnelle Agricoles Nîmes-Rodilhan-CFPPA du Gard, Rodilhan, France; ^5^Centre Technique de l’Olivier, Maison des Agriculteurs, Aix-en-Provence, France; ^6^CNRS-IRD-UPS EDB, UMR 5174, Université Paul Sabatier, Toulouse, France

**Keywords:** *Olea europaea* L., parentage analysis, simple sequence repeat, genetic structure, *ex situ* collection of Porquerolles, genetic resource management, core collection

## Abstract

Molecular characterization of crop genetic resources is a powerful approach to elucidate the origin of varieties and facilitate local cultivar management. Here we aimed to decipher the origin and diversification of French local olive germplasm. The 113 olive accessions of the *ex situ* collection of Porquerolles were characterized with 20 nuclear microsatellites plus their plastid haplotype. We then compared this collection to Mediterranean olive varieties from the Worldwide Olive Germplasm Bank of Marrakech, Morocco. High genetic diversity was observed within local French varieties, indicating a high admixture level, with an almost equal contribution from the three main Mediterranean gene pools. Nearly identical and closely related genotypes were observed among French and Italian/Spanish varieties. A high number of parent–offspring relationships were also detected among French varieties and between French and two Italian varieties (‘Frantoio’ and ‘Moraiolo’) and the Spanish variety (‘Gordal Sevillana’). Our investigations indicated that French olive germplasm resulted from the diffusion of material from multiple origins followed by diversification based on parentage relationships between varieties. We strongly suggest that farmers have been actively selecting olives based on local French varieties. French olive agroecosystems more affected by unexpected frosts than southernmost regions could also be seen as incubators and as a bridge between Italy and Spain that has enhanced varietal olive diversification.

## Introduction

Olive (*Olea europaea* L.) is the iconic fruit crop of the Mediterranean Basin. Archaeological, historical, and genetic studies support a primary olive domestication in the Near East, probably starting during the Chalcolithic period (Kaniewski et al., 2012; Zohary et al., 2012; Besnard et al., 2013b). Then long-distance translocation of varieties followed by admixture events led to secondary multi-local diversification in central and western Mediterranean regions (Terral, 1997; Besnard et al., 2001a; Belaj et al., 2002; Owen et al., 2005; Baldoni et al., 2006; Breton et al., 2008; Diez et al., 2015). Locally adapted varieties have thus been carefully selected by farmers in several Mediterranean areas and the domestication process is still ongoing (Besnard et al., 2001a; Khadari et al., 2003; Khadari et al., 2008; El Bakkali et al., 2013a; Besnard et al., 2018). Selected trees are still both clonally and seed propagated in traditional agroecosystems from different parts of the Mediterranean Basin (Aumeeruddy-Thomas et al., 2017; Besnard et al., 2018), implying a continuing role of sexual reproduction in varietal diversification, with potential contributions from local domesticated, feral, and wild olives. Due to this diversification process, a high frequency of parentage relationships could be expected among varieties, as previously observed within the Spanish olive germplasm (Diez et al., 2015) and in grapevine (Bowers et al., 1999; Lacombe et al., 2013). Furthermore, farmer selection of newly adapted olive trees could be viewed as a key process in agroecosystems under changing climatic and ecological conditions, such as those on the fringe of olive growing areas. But it is still unknown how farmer selection and varietal diversity relate to these changing environmental conditions. Today, in a context of global changes associated with the emergence of pests that threaten olive cropping, especially in southern Europe (e.g. *Xylella fastidiosa*), there is call for the selection of varieties adapted to new environmental conditions (De Ollas et al., 2019). The characterization of olive varieties in any germplasm bank and the elucidation of their origins are thus high priority to ensure efficient use of genetic resources in the future.

In France, olive is traditionally cultivated in southern regions on the rim of the Mediterranean Sea. Major development of olive cultivation was initiated by Phoenicians in Massalia, i.e. present day Marseille, around 2600 BP, while wild olives were already present and some local varieties were also likely cultivated (Terral et al., 2004). More than 100 French olive varieties are currently described based on morphological descriptors (Moutier et al., 2004; Moutier et al., 2011) and molecular markers (Khadari et al., 2003; Khadari et al., 2004). A few of them are considered as main varieties since they are cultivated over relatively large geographical areas, while more than 80 have a restricted distribution range, generally spanning a few townships. This particularly high diversity at the northern limit of the cultivated olive range may partly be the result of recurrent farmer selection of adapted varieties due to relatively frequent frosts that affect local olive germplasm. A significant portion of present varieties (14%) show a maternal origin from the western Mediterranean region, suggesting a local origin (Besnard et al., 2001a; Khadari et al., 2003). Previous studies based on nuclear genetic markers further supported an admixed origin for most French varieties with a prevalent genetic contribution from the eastern Mediterranean (Besnard et al., 2001a; Haouane et al., 2011), as similarly shown in Italian and Tunisian germplasm, respectively on the northern and southern shores of the Mediterranean Sea (Haouane et al., 2011; Belaj et al., 2012; Khadari and El Bakkali, 2018). Such an admixed origin could be seen as a genetic signature of local olive diversification in the central Mediterranean area. However, the local crop diversification process remains unclear, and may involve major progenitors, as shown, for instance, in Andalusian olives (Diez et al., 2015). In addition, it was also shown that cultivars growing in the eastern and western sides of the Rhone valley were differentiated (Khadari et al., 2003), possibly reflecting two pathways of olive cultivar introduction from the Italian and Iberian Peninsulas, respectively.

In the present study, we investigated the cultivated olive diversification process in southern France, with the aim of determining ways to efficiently manage local olive genetic resources. Both nuclear and chloroplast loci were used to characterize the genetic diversity of a set of varieties from the French Olive Germplasm Bank (FOGB) in comparison to the Worldwide Olive Germplasm Bank (WOGB) of Marrakech, Morocco (Haouane et al., 2011; El Bakkali et al., 2013b). We specifically aimed to: (1) assess genetic diversity within the FOGB collection and propose a nested set of French reference varieties representative of total genetic diversity; (2) compare the genetic diversity at two different geographical scales, i.e. local (France) and regional (Mediterranean area); and (3) clarify the origin of French olive germplasm by parentage analyses within and among French and Mediterranean varieties. Our results were examined in light of the diversification process founded on farmer selection within traditional agroecosystems probably hampered by frequent climatic accidents such as frost.

## Material and Methods

### Plant Material

The FOGB includes a total of 113 olive accessions, and is maintained on the island of Porquerolles, near Toulon in southern France (**Table 1**). These accessions are identified with a variety name and/or with tree coordinates in the collection (**Table 1**). Among the 63 accessions identified with a variety name, 14 are considered as being the main French varieties since they are cropped over broad areas compared to minor varieties (22), which have a limited distribution range, generally over a few townships, and to local varieties (27), which are only present in one or two orchards (**Table 1**; Moutier et al., 2004; Moutier et al., 2011).

**Table 1:** List of the 113 French accessions analyzed in the present study classified according to tree coordinates, accession name, code SSR. Accessions showing molecular variants (one or two dissimilar alleles).

Tree coordinates	Accession name	SSR code	Variant code	Reference genotype	Reference variety	Importance of the variety	genetic structure	Chlorotype	French parentage	Mediterranean parentage	CC level
3_03	Aglandau	413	51	3_03	Aglandau	Main	Mosaic	E 1-1	3	1	CC_43_
34_17	Aglandau	414									
33_25	Aglandau	414	51								
32_21	Amellau	459		32_21	Amellau	Secondary	Eastern	E 1-1	1	0	CC_43_
32_03	Araban 06	460		32_03	Araban 06	Local	Mosaic	E 1-1	0	0	CC_22_
31_05	Araban du Var	405									
35_07	Araban du Var	405		35_07	Araban du Var	Secondary	Mosaic	E 1-1	4	2	CC_75_
27_13	Baguet	461		27_13	Baguet	Local	Mosaic	E 1-1	0	0	CC_75_
26_05	Béchude	411		26_05	Béchude	Secondary	Mosaic	E 2-1	2	0	CC_75_
36_02	Béchude	411									
36_22	Béchude	411									
34_04	Bé-dé-Cézé	462		34_04	Bé-dé-Cézé	Secondary	Mosaic	E 1-1	0	0	CC_22_
37_23	Belgentiéroise	463		37_23	Belgentiéroise	Secondary	Mosaic	E 1-1	0	0	CC_43_
1_13	Blanc de Paysac	412		1_13	Blanc de Paysac	Secondary	Eastern	E 1-1	0	0	CC_75_
24_02	Blanc de Paysac	412									
37_11	Blanquetier	464		37_11	Blanquetier	Local	Mosaic	E 3-1	0	3	CC_75_
36_11	Boube	416	47	36_11	Boube	Local	Western	E 1-2	6	30	CC_75_
35_13	Boube	417	47								
15_05	Boube	416									
34_19	Bouteillan	465		34_19	Bouteillan	Main	Mosaic	E 1-1	1	0	CC_22_
35_25	Broutignan	409	50	35_25	Broutignan	Secondary	Mosaic	E 1-1	1	0	CC_22_
35_28	Broutignan	410									
35_22	Broutignan	410	50								
37_21	Brun	466		37_21	Brun	Secondary	Eastern	E 1-1	0	0	CC_75_
32_33	Cailletier	429	3	32_33	Cailletier	Main	Central	E 1-1	6	18	CC_75_
34_31	Cailletier	430	3								
33_31	Cailleton	467		33_31	Cailleton	Local	Mosaic	E 1-1	0	0	CC_22_
33_13	Capelen	468		33_13	Capelen	Local	Mosaic	E 1-1	0	0	CC_43_
15_02	Cayet Rouge	469		15_02	Cayet Rouge	Local	Mosaic	E 1-1	0	2	CC_75_
33_10	Cayet Roux	421	54	33_10	Cayet Roux	Main	Mosaic	E 1-1	2	0	CC_22_
6_11	Cayet Roux	422	54								
37_26	Cayet Roux	423	54								
3_12	Cayon	402		3_12	Cayon	Main	Mosaic	E 2-1	0	0	CC_22_
33_04	Clermontaise	470		33_04	Clermontaise	Secondary	Mosaic	E 1-1	1	0	CC_43_
20_15	Colombale	471		20_15	Colombale	Secondary	Mosaic	E 1-1	2	0	CC_75_
7_14	Corniale	472		7_14	Corniale	Secondary	Mosaic	E 1-1	0	0	CC_43_
8_04	Courbeil	473		8_04	Courbeil	Secondary	Mosaic	E 3-1	1	0	CC_22_
33_07	Cul Blanc	474		33_07	Cul Blanc	Secondary	Mosaic	E 2-1	1	0	CC_75_
7_03	Curnet	475		7_03	Curnet	Secondary	Eastern	E 3-1	0	0	CC_43_
25_03	Darame	476		25_03	Darame	Local	Eastern	E 1-1	3	0	CC_75_
32_20	Dent de Verrat	477		32_20	Dent de Verrat	Local	Central	E 3-1	0	0	CC_22_
19_04	Filayre rouge	478		19_04	Filayre rouge	Local	Eastern	E 1-1	0	0	CC_75_
26_10	Gardisson	479		26_10	Gardisson	Local	Mosaic	E 2-1	0	1	CC_75_
15_07	Grapié	480		15_07	Grapié	Local	Mosaic	E 1-1	0	1	CC_75_
32_09	Grassois	481		32_09	Grassois	Local	Central	E 1-1	2	2	CC_43_
8_16	Gros vert	482		8_16	Gros vert	Local	Central	E 1-1	1	0	CC_22_
17_13	Grossane	415		17_13	Grossane	Main	Mosaic	E 1-1	0	0	CC_75_
37_05	Grossane	415									
35_01	Grosse Noire	483		35_01	Grosse Noire	Local	Eastern	E 1-1	1	0	CC_43_
36_01	Grosse Violette	407	49								
35_04	Grosse Violette	408	49	35_04	Grosse Violette	Secondary	Eastern	E 1-1	0	1	CC_75_
32_15	Linat	484		32_15	Linat	Local	Eastern	E 1-1	1	2	CC_75_
16_08	Lucques	485		16_08	Lucques	Main	Mosaic	E 1-1	0	0	CC_22_
1_17	Malausséna	486		1_17	Malausséna	Local	Mosaic	E 1-1	1	3	CC_22_
35_16	Menudel	487		35_16	Menudel	Secondary	Mosaic	E 1-1	1	0	CC_75_
13_16	Montaurounenque	424	18								
23_10	Montaurounenque	425	18	23_10	Montaurounenque	Secondary	Mosaic	E 2-1	0	0	CC_43_
23_11	Montaurounenque	426	18								
31_15	Moufla	488		31_15	Moufla	Local	Eastern	E 1-1	1	3	CC_75_
34_01	Négrette	427	52								
5_02	Négrette	428	52	5_02	Négrette	Main	Mosaic	E 1-1	5	0	CC_75_
6_12	Olivière	403		6_12	Olivière	Main	Mosaic	E 3-1	3	0	CC_75_
10_03	Petit Ribier	418	27	10_03	Petit Ribier	Main	Central	E 1-1	3	4	CC_75_
16_14	Petit Ribier	419	27								
11_07	Petit Ribier	420	27								
22_02	Petite Noire	489		22_02	Petite Noire	Secondary	Mosaic	E 1-1	1	0	CC_75_
36_07	Petite Violette	490		36_07	Petite Violette	Local	Mosaic	E 1-1	1	0	CC_22_
17_07	Picholine	404		17_07	Picholine	Main	Mosaic	E 2-1	3	0	CC_43_
30_15	Pigale	491		30_15	Pigale	Local	Mosaic	E 2-1	1	0	CC_75_
6_06	Rascasset	492		6_06	Rascasset	Local	Mosaic	E 1-1	0	0	CC_75_
6_01	Reymet	493	53	6_01	Reymet	Secondary	Central	E 1-1	1	1	CC_22_
35_10	Ronde de VDB	494		35_10	Ronde de VDB	Local	Mosaic	E 1-1	2	1	CC_75_
4_14	Rougette de l’Ardèche	495		4_14	Rougette de l’Ardèche	Main	Mosaic	E 2-1	1	0	CC_43_
36_31	Rougette de Pignan	496		36_31	Rougette de Pignan	Secondary	Mosaic	E 1-1	0	0	CC_22_
36_16	Rougette du Gard	497		36_16	Rougette du Gard	Secondary	Mosaic	E1.4	1	0	CC_43_
33_16	Salonenque	498		33_16	Salonenque	Main	Eastern	E 1-1	0	2	CC_43_
36_19	Sauzin Vert	499		36_19	Sauzin Vert	Local	Mosaic	E1.4	1	1	CC_75_
2_05	Tanche	500		2_05	Tanche	Main	Eastern	E 1-1	0	1	CC_75_
22_08	Taulelle	501		22_08	Taulelle	Local	Mosaic	E 1-1	0	0	CC_22_
31_09	Tripue	502		31_09	Tripue	Local	Mosaic	E 1-1	0	0	CC_22_
34_13	Verdale 13	503		34_13	Verdale 13	Secondary	Eastern	E 1-1	0	0	CC_75_
10_10	Verdanel	504		10_10	Verdanel	Local	Mosaic	E 1-1	1	0	CC_75_
22_14	Vilette	505		22_14	Vilette	Local	Mosaic	E 1-1	2	0	CC_75_
10_04	10_04	431		10_04			Mosaic	E 1-1	2	0	
10_09	10_09	432		10_09			Mosaic	E 1-1	0	0	
13_12	13_02	433		13_12			Mosaic	E 1-1	0	0	
14_17	14_17	434		14_17			Mosaic	E 1-1	1	0	
16_04	16_04	406		16_04			Mosaic	E 1-1	0	0	
18_16	18_16	435		18_16			Mosaic	E 1-1	0	0	CC_22_
19_02	19_02	436		19_02			Mosaic	E 1-1	1	0	CC_43_
20_11	20_11	437		20_11			Mosaic	E 1-1	2	1	CC_43_
23_04	23_04	438		23_04			Mosaic	E 1-1	1	0	
24_09	24_09	439		24_09			Eastern	E 1-1	0	0	
32_25	32_25	440		32_25			Mosaic	E 1-1	0	0	CC_43_
33_00	33_00	441		33_00			Mosaic	E 3-1	0	0	CC_22_
33_02	33_02	442		33_02			Mosaic	E 3-1	0	0	
33_19	33_19	443	33	33_19			Mosaic	E 1-1	1	0	CC_43_
33_22	33_22	444		33_22			Mosaic	E 1-1	0	0	CC_43_
33_32	33_32	445		33_32			Mosaic	E 1-1	3	0	
34_07	34_07	406									
34_10	34_10	446		34_10			Mosaic	E 1-1	4	0	
34_22	34_22	447		34_22			Central	E 1-1	1	1	CC_43_
34_25	34_25	448		34_25			Mosaic	E 1-1	1	0	CC_22_
34_28	34_28	449		34_28			Mosaic	E 1-1	2	2	
35_19	35_19	450		35_19			Mosaic	E 1-1	3	0	CC_43_
35_31	35_31	451		35_31			Mosaic	E 1-1	0	0	CC_22_
36_04	36_04	452		36_04			Eastern	E 1-1	2	2	
36_25	36_25	453		36_25			Mosaic	E 1-1	5	2	
36_28	36_28	454		36_28			Mosaic	E 3-3	1	0	
37_02	37_02	455		37_02			Mosaic	E 1-1	2	2	
37_25	37_25	456		37_25			Central	E 1-1	1	0	CC_22_
9_01	9_01	457		9_01			Mosaic	E 1-1	2	0	
9_07	9_07	458		9_07			Mosaic	E 1-1	1	0	

Main varieties: planted in large areas.

Secondary varieties: present in a restricted distribution range, generally over a few townships.

Local varieties: only present in one or two orchards.

Genotypes of French accessions were compared to those of other varieties collected throughout the Mediterranean Basin. Four hundred and sixteen accessions from 13 Mediterranean countries that are maintained in the World Olive Germplasm Bank of Marrakech (WOGB; **Supplementary Table S1**) were analyzed. Mediterranean varieties conserved in the WOGB collection are classified in three gene pools based on both the country origin and genetic structure, i.e. East (mostly from Cyprus, Egypt, Lebanon, and Syria), West (mostly from Morocco, Spain, and Portugal), and Central (mostly from Algeria, Italy, Slovenia, Croatia, Tunisia, and Greece; Haouane et al., 2011; El Bakkali et al., 2013b).

### Datasets

Twenty microsatellite nuclear loci (SSR) were used for genotyping accessions of both FOGB and WOGB (**Table 2**), as described by El Bakkali et al. (2013b). These markers were selected based on their clear amplification, high polymorphism, and reproducibility, as reported by Trujillo et al. (2014). Alleles were carefully scored twice independently by two researchers. Genotyping of accessions with a specific allele (i.e. observed only once) was systematically repeated to ensure its occurrence. Plastid DNA (*cpDNA*) variations were also characterized using 39 markers, including 32 cpSSR loci, five indels (insertions/deletions), and two single nucleotide polymorphisms (SNPs), as described by Besnard et al. (2011).

**Table 2:** Genetic parameters of the 20 SSR loci in both FOGB (92 genotypes) and WOGB (311) collections.

N	Loci	FOGB						WOGB			
		Size	Na	Npa	He	Ho	PIC	Size	Na	He	Ho
**1**	DCA01^a^	203–268	7 (1)^1^	2	0.568	0.620	0.520	203–274	19	0.641	0.736
**2**	DCA03^a^	229–250	7		0.841	0.935	0.814	227–263	14	0.854	0.891
**3**	DCA04^a^	128–192	20 (1)	6	0.875	0.674	0.856	116–198	34	0.856	0.666
**4**	DCA05^a^	189–209	11		0.614	0.685	0.587	189–211	12	0.513	0.511
**5**	DCA08^a^	123–154	15	6	0.795	0.891	0.765	123–164	21	0.837	0.945
**6**	DCA09^a^	160–207	18 (1)	6	0.873	0.913	0.855	160–217	24	0.889	0.952
**7**	DCA11^a^	125–179	11	5	0.788	0.913	0.752	125–199	24	0.830	0.868
**8**	DCA14^a^	168–186	9	1	0.636	0.641	0.604	166–190	15	0.712	0.740
**9**	DCA15^a^	242–265	4		0.519	0.489	0.443	242–265	7	0.656	0.695
**10**	DCA16^a^	121–175	10	2	0.815	0.615	0.786	121–230	35	0.879	0.952
**11**	DCA18^a^	162–182	10	1	0.815	0.870	0.788	154–188	17	0.846	0.916
**12**	GAPU59^b^	206–226	7	2	0.585	0.565	0.546	206–238	11	0.623	0.592
**13**	GAPU71A^b^	207–239	5	1	0.325	0.337	0.296	205–255	16	0.476	0.555
**14**	GAPU71B^b^	116–141	6	1	0.801	0.924	0.765	116–144	8	0.807	0.900
**15**	GAPU101^b^	181–215	8		0.851	0.967	0.828	181–217	13	0.858	0.945
**16**	GAPU103A^b^	133–188	14	3	0.827	0.867	0.802	133–194	26	0.862	0.781
**17**	EMO03^c^	201–215	11 (1)	4	0.767	0.707	0.727	201–215	13	0.806	0.807
**18**	EMO90^c^	180–193	5		0.710	0.837	0.666	180–208	9	0.658	0.672
**19**	UDO-017^d^	152–168	6		0.784	0.804	0.745	144–172	9	0.777	0.820
**20**	UDO-036^d^	140–164	7	2	0.683	0.739	0.625	138–166	12	0.731	0.706
	**Mean**		**9.55**		**0.723**	**0.749**	**0.688**		**16.95**	**0.755**	**0.782**
	**Total**		**191 (4)**	**42**					**339**		

^1^between brackets: number of specific alleles compared to the WOGB collection.

^a^ Sefc et al., 2000, ^b^ Carriero et al., 2002, ^c^ De la Rosa et al., 2002, ^d^ Cipriani et al., 2002.

Number of alleles (Na), number of private alleles (Npa), expected (He) and observed heterozygosity (Ho), polymorphism information content (PIC).

## Data Analysis

### Genetic Diversity and Structure

The number of alleles per locus (*Na*), expected (*He*; Nei, 1987) and observed heterozygosity (*Ho*), and polymorphism information content (PIC) were estimated using the Excel Microsatellite Toolkit v.3.1 (Park, 2001).

A binary matrix containing only distinct French genotypes was built, using alleles scored as present (1) or absent (0) to assess genetic relationships within the FOGB collection. This matrix was used to construct a dendrogram based on Dice’s similarity index (Dice, 1945) and the UPGMA algorithm with the NTSYS v2.02 software package (Rohlf, 1998).

The French (FOGB) and Mediterranean (WOGB) collections were compared based on different criteria: (1) genetic parameters such as the allele number (*Na*), expected and observed heterozygosity (*He* and *Ho*); (2) the distribution of pairwise genetic distances between cultivars using the index of Smouse and Peakall (1999) in GENALEX 6 program (Peakall and Smouse, 2006); (3) the allelic richness (*Ar*) using the ADZE program (Szpiech et al., 2008); (4) a principal coordinate analysis (PCoA) implemented in DARWIN 5.0.137 (Perrier et al., 2003) using the simple matching coefficient to describe relationships between genotypes based on the spatial distribution of the two first coordinate axes; and (5) the genetic structure within both collections using the model-based Bayesian clustering approach implemented in STRUCTURE v.2.2 (Pritchard et al., 2000) according to the parameters described in Haouane et al. (2011). Regarding the genetic structure, the reliability of the number of clusters (*K*) was checked using the *ad hoc* Δ*K* measure (Evanno et al., 2005) with the R program, whereas the similarity index between different replicates for the same *K* clusters (*H′*) was calculated using the CLUMPP v1.12 program (Jakobsson and Rosenberg, 2007).

### Parentage Analysis

Parentage analyses were based on nuclear SSR data and aimed at detecting putative parent–offspring relationships among French varieties, as well as between these latter and varieties from the whole Mediterranean Basin. A putative parent–offspring pair is defined as any pair of individuals that share alleles across all loci and contain all true and false parent–offspring pairs (Jones et al., 2010). Indeed, the probability of two unrelated genotypes sharing alleles by chance at all loci is not trivial, especially for a large set of pairwise comparisons with a limited number of molecular markers. A key challenge addressed in our analyses was to correctly identify the true parent–offspring pairs within a dataset, while simultaneously excluding pairs that could potentially have shared alleles by chance. Considering the large panel of examined varieties without any available information on parentage relationships, pedigree reconstruction based on parental pair assignment may be not robust, as in cases when one parent is already known (Jones et al., 2010), and the probability of detecting false parent–offspring pairs would thus need to be assessed. Here, in a first step, we conducted parentage analyses through a “single-parent search” (Jones et al., 2010) in order to identify putative parent–offspring pairs. Second, based on these results, we used parental pair assignment to construct pedigree among varieties.

For single-parent searches, we used a complete exclusion approach and two parentage assignment approaches where by the single most likely parent was chosen from a group of non-excluded candidate parents based on a likelihood method or on Bayesian posterior probability of a: (*i*) First, we used the exclusion-based method with the PARFEX v.1.0 macro (Sekino and Kakehi, 2012). This simple method examines genotype incompatibilities between offspring and parents based on Mendelian inheritance rules. A parentage relationship is established if a single parent of offspring remains non-excluded from a parental pool considering 0 or 1 mismatching allele at a single locus; (*ii*) we then used the likelihood-based method (Gerber et al., 2000) available in the PARFEX v.1.0 macro. This parentage inference relies on the difference in the log-likelihood ratio (LOD) between related and unrelated relationships. To define a threshold (LODc) to accept/reject possible parentage relationships (single parent), offspring were simulated using the allelic frequencies (L_obs_) observed in our datasets and a random sampling of alleles (L_rand_), while taking into account the genotypic error rate for random replacement of simulated genotypes at each marker (e_sim_) and for LOD calculations (e_calc_). Simulations were conducted using 1% error rates for e_calc_ and e_sim_, 200 parents, and 10,000 offspring. The LODc was defined by the intersection of the distribution of L_obs_ and L_rand_; (*iii*) Lastly, based on the exclusion-Bayes’ theorem method (Christie, 2010) using SOLOMON package in the R program (Christie et al., 2013), the posterior probability of false parent–offspring pairs (among all pairs that share at least one allele across all loci) was assessed in a dataset to determine whether all putative parent–offspring pairs could be accepted with strict exclusion. The probability of observing shared alleles between unrelated individuals was calculated using 1,000 simulated datasets and 50,000,000 simulated genotypes. Finally, parentage inferences of each French genotype were considered as reliable when validated by the three approaches. By detecting single parent–offspring relationships, the identity of parents and offspring of each putative pair could not be determined. Networks of parent–offspring relationships were plotted with the “i*graph*” package in R environment (Csardi and Nepusz, 2006).

For parental pair assignments, putative parent–offspring relationships detected with the three previous approaches were re-used. We used the likelihood-based method (Gerber et al., 2000) to assess this panel of relationships because it appears to be the most conservative approach compared to the exclusion-Bayes’ theorem method (see *Results*).

### Core Collection Sampling

For agronomic experiments and breeding programs, it may be necessary to define sets of cultivars representative of French cultivated olive germplasm. French core collections were thus constructed from the FOGB collection according to the two-step method described by El Bakkali et al. (2013b). Nested core collections were constructed by combining two approaches implemented in the CoreHunter (Thachuk et al., 2009) and Mstrat (Gouesnard et al., 2001) programs. First, an initial core collection capturing total allelic diversity was constructed with Mstrat to estimate the sample size necessary to capture all observed alleles. Then CoreHunter with the “*Sh strategy*” was run with half of the initial constructed core collection in order to select a primary local core collection with the lowest number of accessions. This primary core collection was used as a kernel in Mstrat to capture the remaining alleles and 50 independent core collections were proposed.

## Results

### Characterization of French Olive Germplasm and Definition of Reference Genotypes per Variety

**Figure 1:**
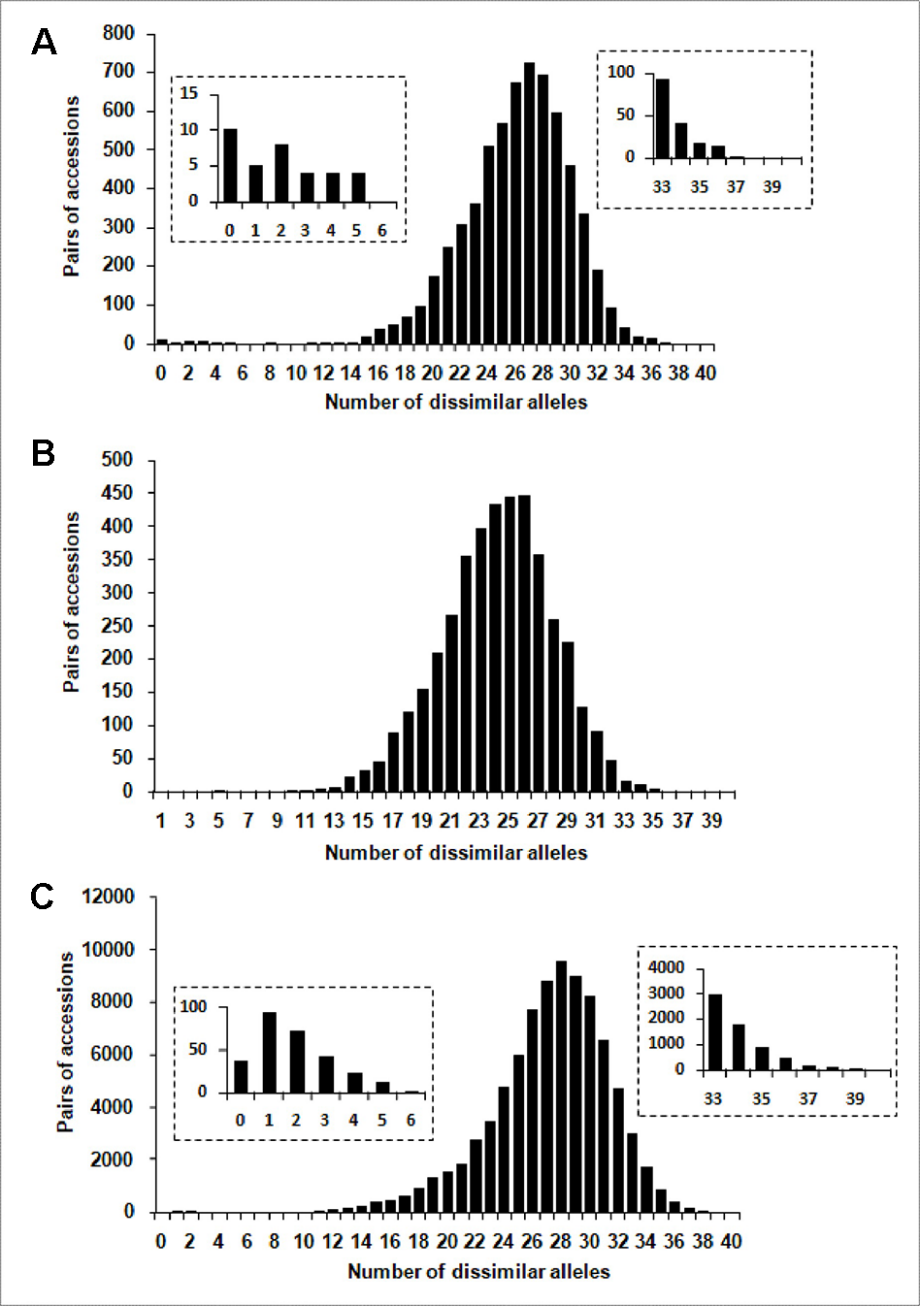
Distribution of the number of dissimilar alleles for all pairwise comparisons for: **(A)** the 113 accessions in the FOGB collection, **(B)** the 92 French genotypes, and **(C)** the 416 accessions in the WOGB collection.

**Figure 2:**
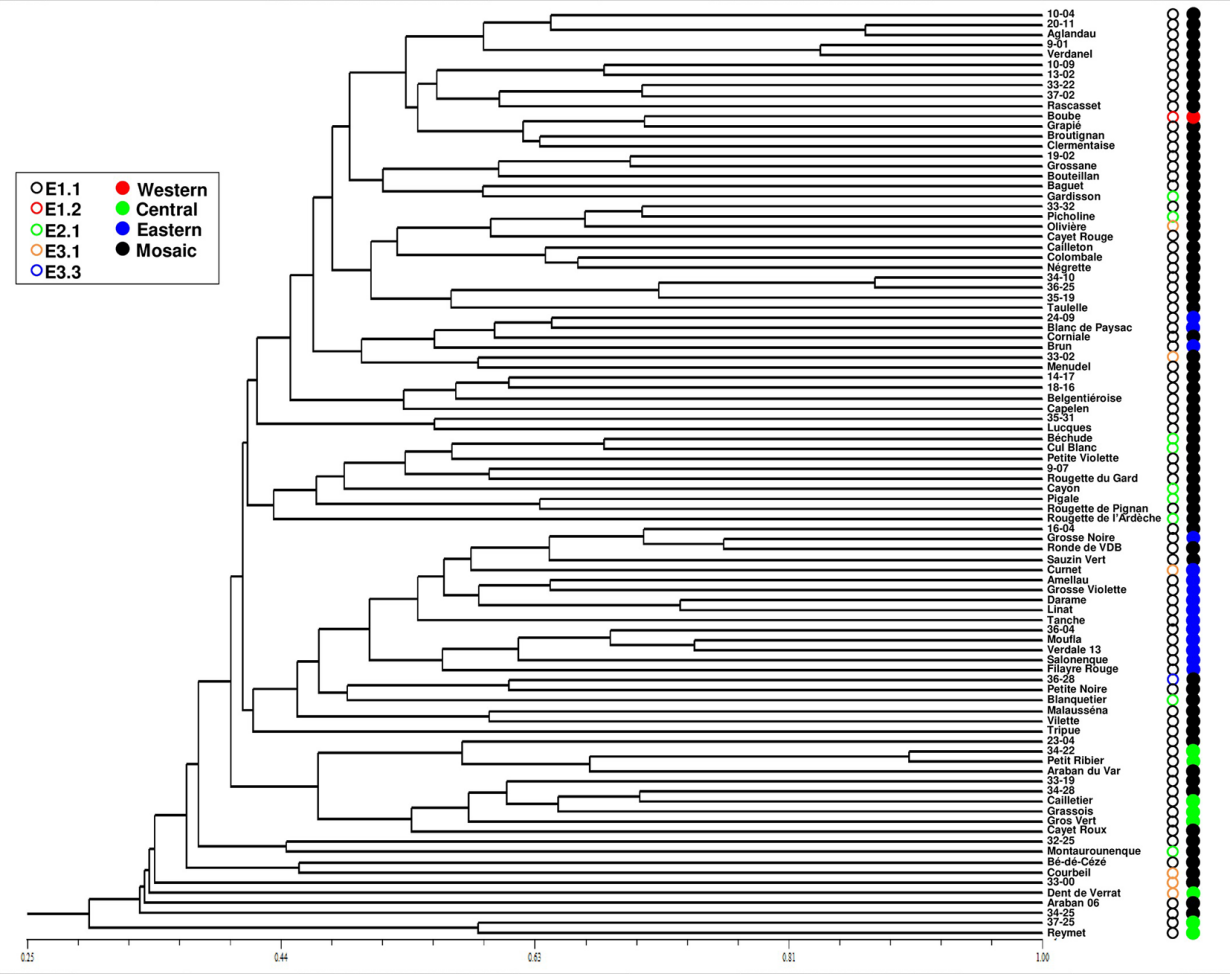
Classification of the 92 genotypes identified among the 113 accessions in the FOGB collection using 20 SSR loci. Maternal lineage and assignment to each gene pool are indicated by circles. The closest genotypes are distinguished by five dissimilar alleles and most genotypes in the FOGB collection were shown as admixed and carrying the E1.1 plastid haplotype.

One hundred and four distinct genetic profiles were obtained among the 113 accessions of the FOGB based on 20 SSR nuclear loci (**Table 1**). Among the 6328 pairwise comparisons, 10 were identical (0.16%), 13 (0.19%) were closely related and differing by one or two dissimilar alleles, whereas the remaining pairs were distinguished by three to 37 dissimilar alleles (**Figure 1A**). Closely related SSR profiles with one or two dissimilar alleles were considered as putative molecular variants resulting from somatic mutations and were thus classified as a single genotype. This was the case for ancient varieties such as ‘Boube’ or ‘Négrette’ and also for major varieties, such as ‘Aglandau’ or ‘Cailletier’, which are cultivated over broad geographic areas (**Supplementary Table S2**). The SSR profile considered as the reference genotype of the variety was chosen based on the high frequency of trees under the same molecular profile (**Table 1** and **Supplementary Table S2**). Hence, a total of 92 genotypes was defined among the 113 accessions analyzed and the most closely related pairs were ultimately distinguished by five dissimilar alleles; e.g. ‘Petit Ribier’ and ‘34-22’ (**Figures 1B** and **2**; **Table 1**).

According to the methodology proposed by Khadari et al. (2003), a total of 63 varieties were validated as reference varieties by checking the morphological traits of olive stones and SSR profiles of several trees originating from different nurseries and orchards (**Table 1**). For instance, six trees of the ‘Cailletier’ variety from distinct origins were analyzed to define the reference genotype (Moutier et al., 2004). Similarly, a total of 15 and 18 trees from different nurseries and orchards were analyzed to validate the reference genotypes of the ‘Petit Ribier’ and ‘Négrette’ varieties, respectively (Moutier et al., 2004; Moutier et al., 2011). The remaining 30 accessions, classified by tree coordinates in the germplasm collection, are currently being validated to determine the reference genotype of each variety according to the methodology described here (**Table 1**).

### Nuclear and Plastid DNA Polymorphism

Considering the 92 genotypes of the FOGB, a total of 191 alleles were revealed with an average of 9.55 alleles/locus (**Table 2**). Among the 191 alleles detected, 42 (22%) were observed once. For each SSR locus, PIC values ranged from 0.296 at the GAPU71A locus to 0.856 at the DCA04 locus (mean 0.688). Only three out of the 20 loci used were able to discriminate between the 92 genotypes revealed among the 113 accessions analyzed, i.e. DCA04, DCA09, and GAPU101 (**Supplementary Table S3**).

The use of 39 chloroplastic loci revealed the presence of six chlorotypes in the French olive germplasm. As expected (see Besnard et al., 2013b), the most frequent chlorotype was E1.1 (79.4%). One of the five other haplotypes was detected once, i.e. E3.3 in the accession referred to as ‘36-28’ (**Table 1**; **Figure 2**).

### Comparison Between French and Mediterranean Olive Germplasm

#### Characterization and Pairwise Comparison Between the Two Germplasm Collections

Based on pairwise analysis of the WOGB with 20 nuclear loci, 404 single SSR profiles (min. 1 dissimilar allele) were identified among the 416 Mediterranean olive accessions. Among the 86320 pairwise comparisons, 36 were identical (0.04%), 166 (0.19%) were closely related (differing by one or two dissimilar alleles), whereas the remaining were distinguished by 3 to 40 dissimilar alleles (**Figure 1C**). Similar to the FOGB collection (see above), accessions showing identical profiles and those with one or two dissimilar alleles (molecular variants) were considered as belonging to the same genotype, leading to a total of 311 distinct genotypes among the 416 accessions analyzed (**Supplementary Table S1**).

Pairwise comparisons between the two collections revealed that eight French accessions were identical or closely related to 28 Mediterranean varieties (**Table 3**). Eighteen out of the 28 varieties originated from Italy, four from Lebanon, whereas the six remaining varieties were from Algeria (2), Spain (1), Cyprus (1), Greece (1), and Morocco (1).

**Table 3:** Cases of genetically similar or close varieties found in the identification process between the FOGB and the WOGB based on 20 SSR loci.

	French variety (FOGB)	Mediterranean variety (WOGB)	Number of dissimilar alleles	Origin
1	^#^Boube	Gordal Sevillana^$^	2	Spain
		Santa Caterina	2	Italy
		Aguenaou	2	Algeria
2	^#^Cailletier	Arancino*	1	Italy
		Augellina*	1	Italy
		Correggiolo di pallesse*	1	Italy
		Frantoio*^$^	1	Italy
		Larcianese*	1	Italy
		Razzo*	1	Italy
		Puntino*	1	Italy
		San Lazzaro	2	Italy
		Baladi Ain	1	Lebanon
		Jlot	2	Lebanon
		BaladiTawil*	1	Lebanon
		Fakhfoukha	2	Morocco
3	^#^Petit Ribier	Filare	2	Italy
		Moraiolo^$^	1	Italy
		Tondello	2	Italy
		Alethriko	2	Cyprus
4	Cayon	Rougette de Mitidja	0	Algeria
5	Olivière	Kalokerida	0	Creece
6	Picholine	AbouChawkeh	0	Lebanon
7	Reymet	Ciliegino*	1	Italy
		Rosino*	1	Italy
		Rossellino*	1	Italy
		Pesciatino*	1	Italy
8	^#^33-19	Leccino*^$^	2	Italy
		Gremignolo*	2	Italy

*Accessions showed similar in WOGB collection. ^#^French variety similar to foreign one and the reference variety.

^$^The reference variety for the case of similarity between French and Foreign varieties.

#### SSR Polymorphism and Genetic Diversity

The 92 genotypes identified in the FOGB collection were used for comparison with the distinct WOGB genotypes. Among the 191 alleles revealed in the FOGB collection, 187 were present in the WOGB genotypes (339 alleles; **Table 2**). Only four alleles were detected in the French germplasm (**Table 2**); DCA04-172 in ‘Amellau’, DCA01-223 in ‘Clermontaise’, ‘Lucques’, ‘Tripue’, ‘35-31’, and ‘Rougette de Pignan’, DCA09-175 in ‘Clermontaise’, and EMO03-204 in ‘Rougette de Pignan’. Their presence was checked following a second genotyping.

**Figure 3:**
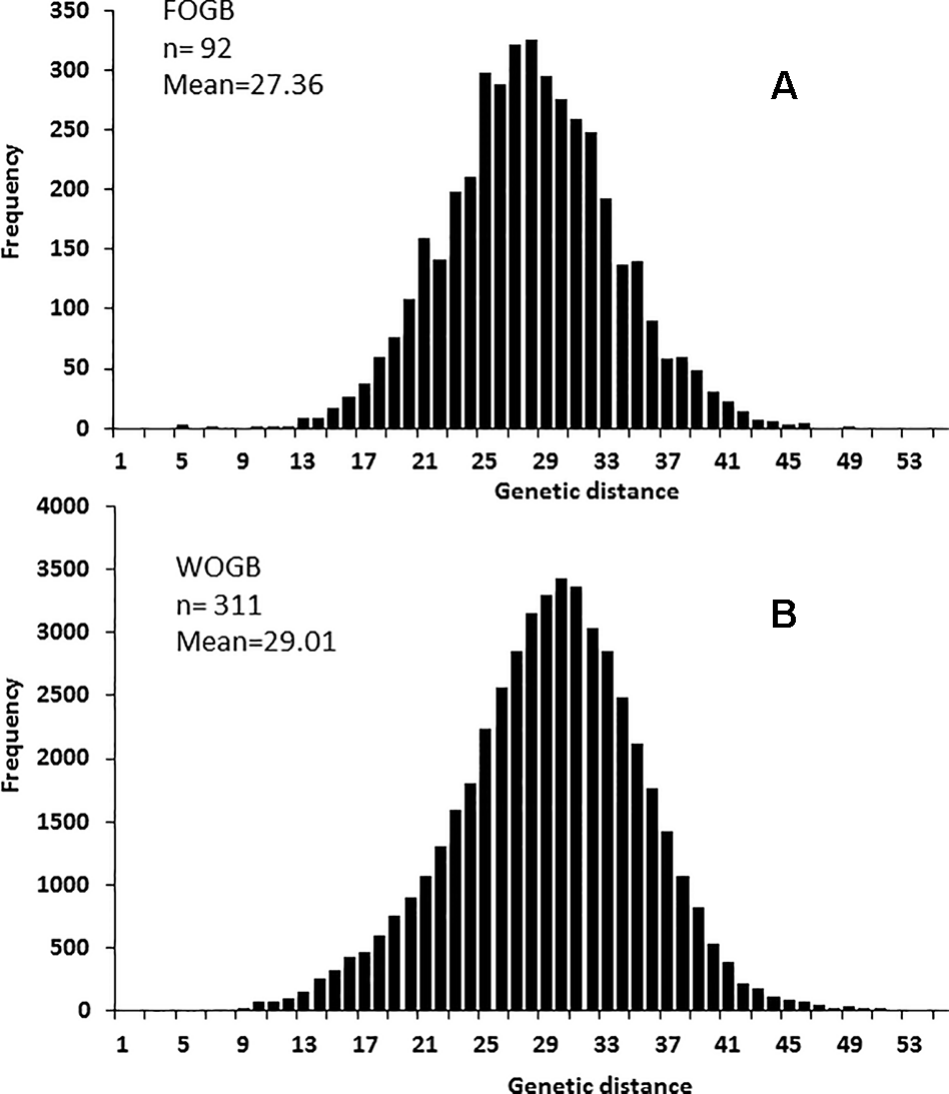
Pairwise distribution of genetic distances using 20 SSR in: **(A)** the FOGB collection, and **(B)** the WOGB collection.

A significant difference in allelic richness computed at a standardized G value of 92 individuals (Kruskal–Wallis test; P-value = 0.032; **Supplementary Table S4**) was observed between the FOGB and WOGB collections. However, the expected heterozygosity (*He*) between the two collections was not significantly different (Kruskal–Wallis test, P-value = 0.317). A similar pairwise genetic distance pattern [index of Smouse and Peakall (1999)] was observed in both FOGB and WOGB (**Figure 3**): ranging from 3 to 55 (with a mean of 29.01) in WGOB, and from 3 to 49 (mean of 27.36) in FOGB.

#### Genetic Structure

**Figure 4:**
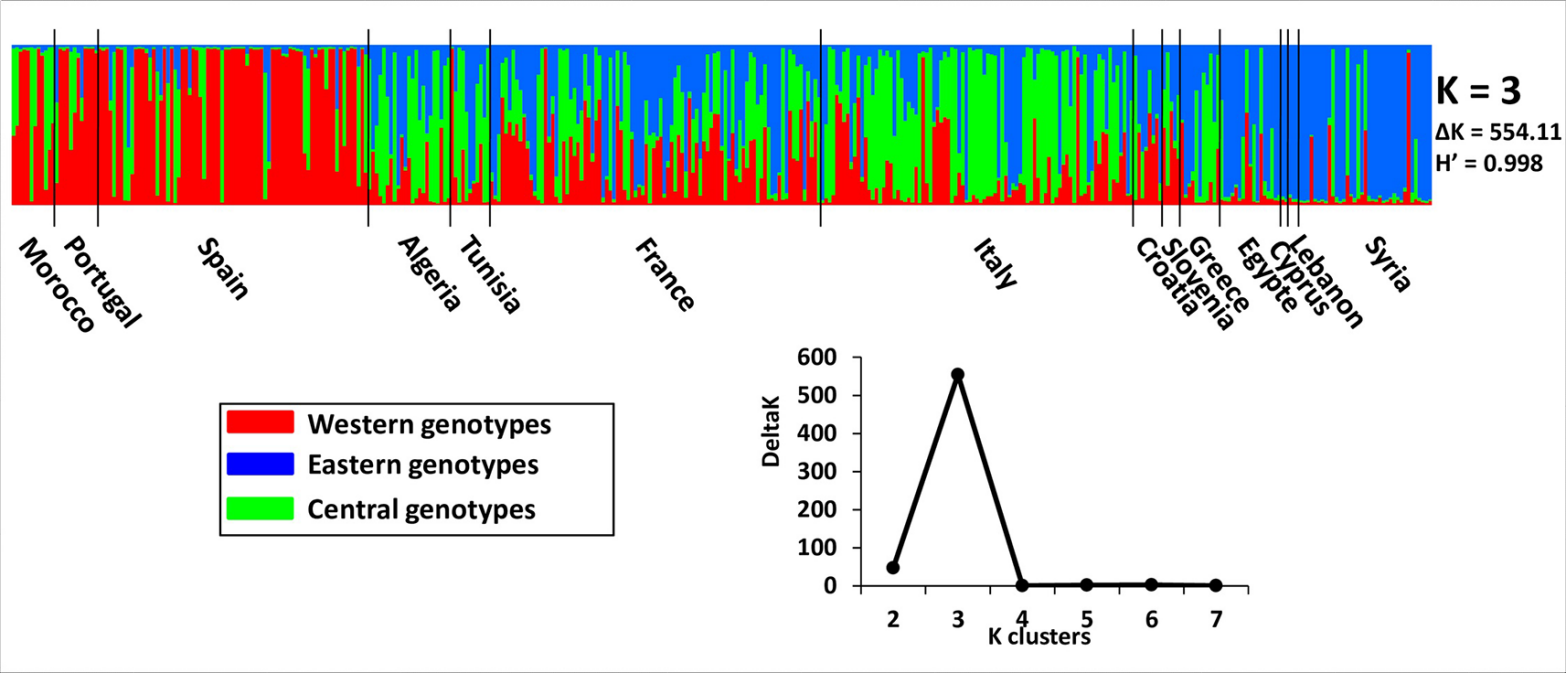
The most probable genetic structure model using the program at *K* = 3 for 395 distinct genotypes from both collections. *H′* represents the similarity coefficient between runs for each *K*, and Δ*K* represents the *ad hoc* measure of Evanno et al. (2005).

Admixture model-based Bayesian clustering was performed on both datasets, with a total of 395 distinct genotypes from both collections. According to Δ*K* and *H′*, *K* = 3 was the most probable genetic structure model (Δ*K* = 554.11 and *H′* = 0.998; **Figure 4** and **Supplementary Figure S1**). Among the 92 French genotypes, 15, 8, and 1 were assigned, with a membership probability of Q ≥ 0.80, to East, Central, and West gene pools, respectively; whereas, 68 (73.9%) genotypes were assigned to more than one group, with Q < 0.80 (**Table 1** and **Supplementary Table S6**).

**Figure 5:**
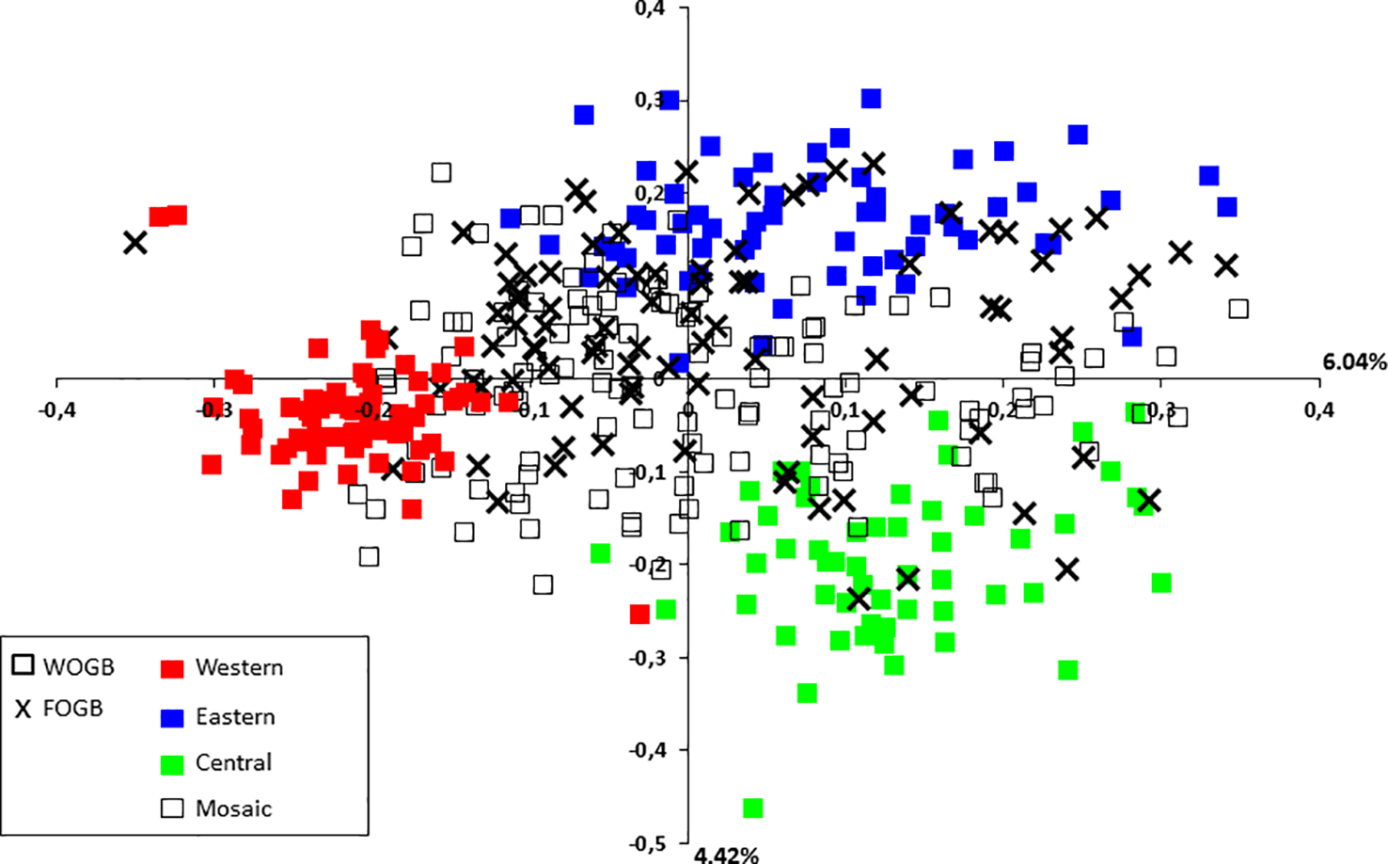
Two-dimensional distribution of the principal coordinates analysis (PCoA) for the 395 distinct genotypes from both collections. Gene pools as identified by for genotypes of the WOGB collection are shown by different colors.

A principal coordinate analysis (PCoA) was conducted and the findings were plotted according to genetic groups, as identified by the program. The first two principal axes explained 10.46% of the total genetic variance (**Figure 5**). French cultivars were classified within the main total diversity range observed in WOGB. The majority of French genotypes were classified in the Mosaic Mediterranean group (Q < 0.80; **Supplementary Table S6**).

### Parentage Relationships Between French and Mediterranean Olive Cultivars

Relationship analyses were conducted using genotypes from the FOGB and WOGB collections with more than two dissimilar alleles. The eight genotypes of the WOGB detected to be identical or genetically close to those of FOGB were also excluded (**Table 3**). Finally, 92 and 303 genotypes from FOGB and WOGB, respectively, were included in the analyses (**Table 1** and **Supplementary Table S1**).

**Figure 6:**
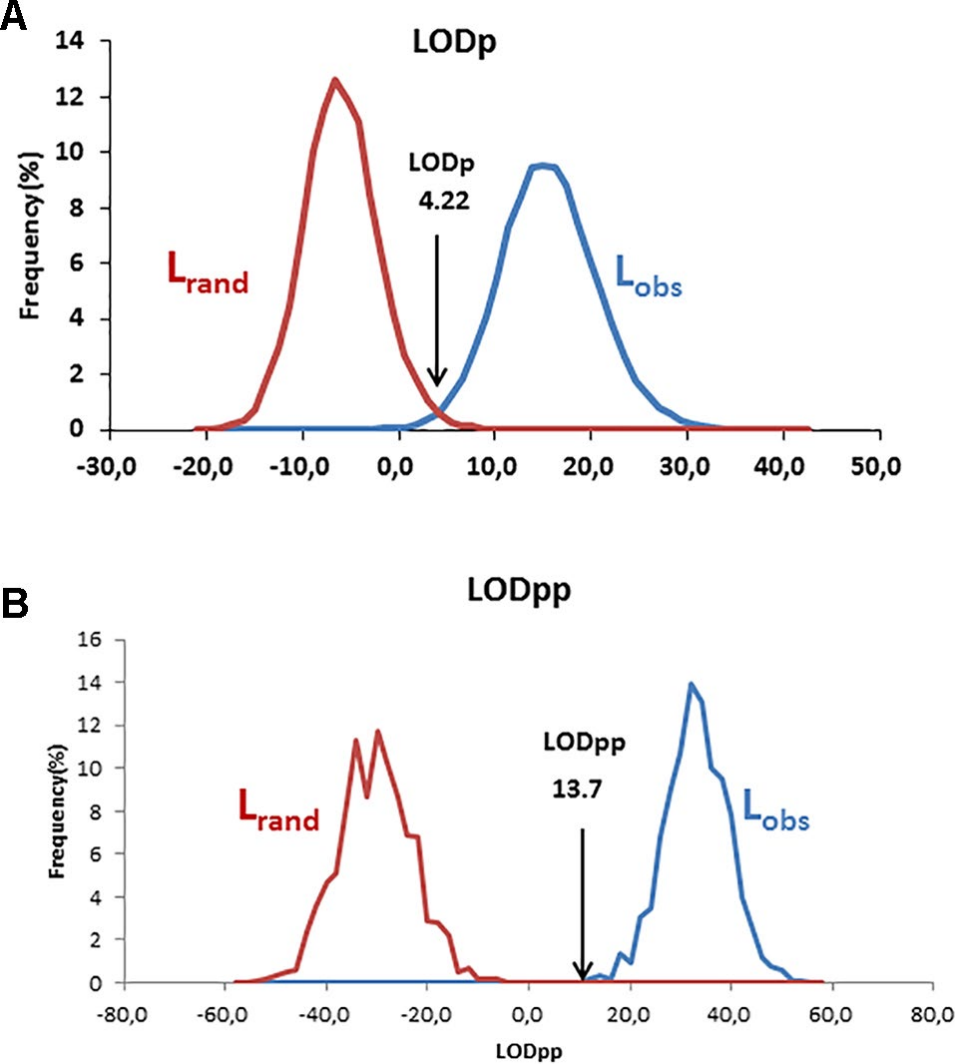
Distribution and intersection between L_obs_ and L_rand_ using v1.0 macro software with the log-likelihood ratio (LOD) method using a single parent search **(A)** and paired parental search **(B)**.

**Figure 7:**
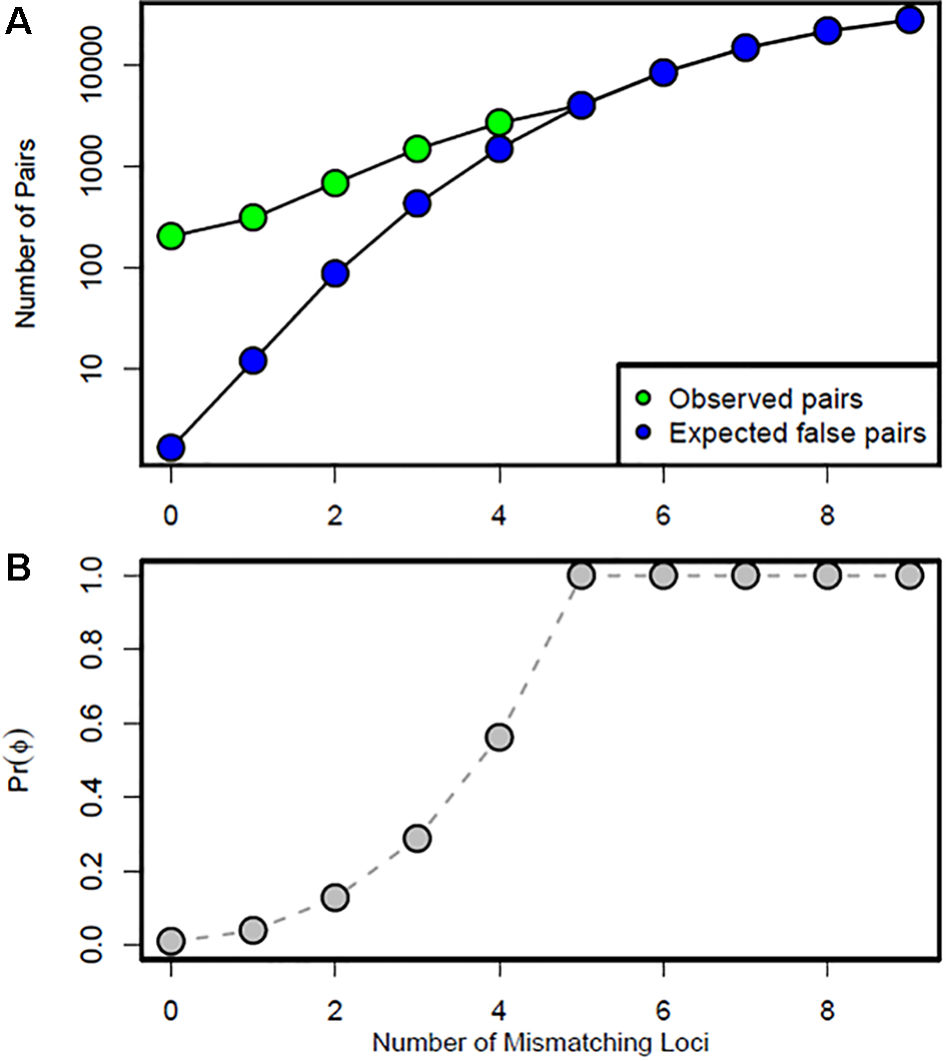
Data simulation results: **(A)** number of observed putative (green points) and expected false (blue points) parent–offspring pairs in the test datasets, and **(B)** Bayesian prior probability Pr(Phi) according to the number of mismatching loci. Any pair that mismatched at one locus would have a 0.03547 probability of occurring by chance.

Using the log-likelihood ratio (LOD) method, the LODc was estimated as the intersection between L_rand_ and L_obs_. A threshold LOD at 4.22 allowed us to define the success rate in detecting true parent–offspring relationships at 97.7% (**Figure 6A**). We thus applied this value in parentage testing for the observed data. Otherwise, the exclusion-Bayes’ theorem method indicated a posterior probability that any pair of genotypes shared at least one allele across all loci (no mismatching across all loci) by chance is Pr(Phi) = 0.00319, while it was 0.03547 for a false parent–offspring pair with a mismatch at one locus (**Figure 7**). Since we could not exclude the possibility that there might have been a few errors in our dataset (including somatic mutations and null alleles), we used the threshold <0.03547 as a cutoff for identifying putative parents.

The putative parent–offspring relationships with the highest probability were observed with the exclusion method (431 parent–offspring pairs), while the Bayesian and the LOD methods gave rise to the lowest number (368 and 239, respectively; **Supplementary Table S5**). The number of French genotypes with a putative parent–offspring relationship differed between methods: 81 genotypes for the Bayesian-based method, 75 for the exclusion method, and 68 for the LOD method.

**Figure 8:**
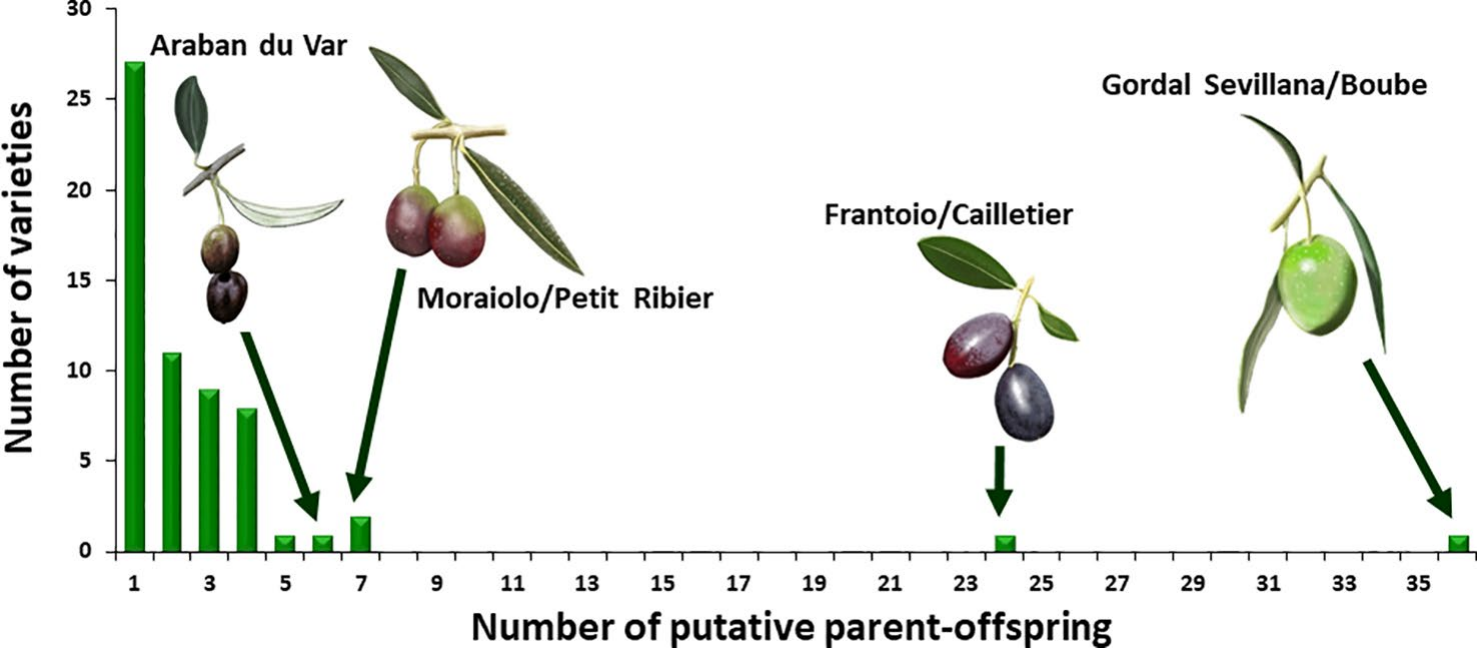
Histogram of the number of putative parents-offsprings observed for French varieties.

For the French varieties, a total of 193 putative parent–offspring pairs were identified when validated by the three approaches. Among these, 101 were detected within the French germplasm since 51 French genotypes (55.4%) were found to have reliable parentage relationships with French varieties only (**Table 1** and **Supplementary Table S5**). Two French varieties showed a particularly high number of putative parent–offspring relationships, i.e. ‘Boube’ and ‘Cailletier’, with 36 and 24, respectively (**Figure 8**), but most of their parentage relationships were established with non-French varieties (30 and 18 putative parent–offspring pairs for ‘Boube’ and ‘Cailletier’, respectively). For other French varieties, the number of putative parent–offspring relationships varied from one to six within the French germplasm, and from one to four between French and other Mediterranean varieties (**Table 1** and **Supplementary Table S5**).

**Figure 9:**
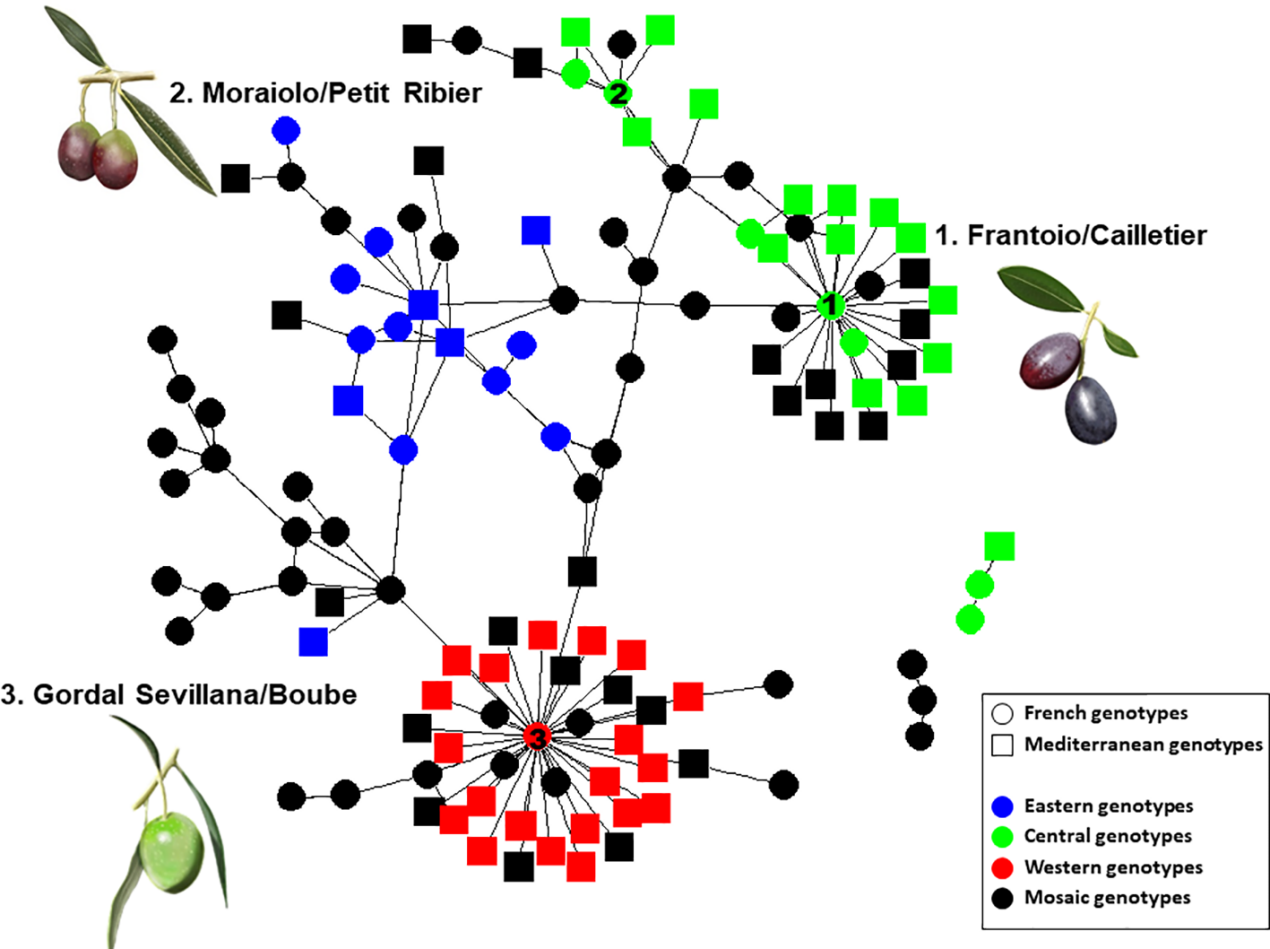
Network of French and Mediterranean varieties showing parentage relationships according to different gene pools as identified by the program.

Most parent–offspring relationships identified belonged to the same genetic group (**Figure 9** and **Supplementary Figure S2**; **Table 4**). Varieties from 11 countries, except Cyprus, Egypt, and Lebanon, showed at least one putative parent–offspring relationship with a French variety. When comparing the origins of these cultivars, we found that varieties from France, Italy, and Spain had the highest proportion of parentage relationships (**Table 4**). Out of 115 Mediterranean cultivars, 51 (44.3%), 28 (24.3%), and 18 (15.6%) varieties, respectively, came from France, Italy, and Spain (**Table 4**). These results underline the importance of parentage relationships within the French germplasm and between French, Spanish, and Italian varieties.

**Table 4:** Numbers and proportion of varieties per countries showing parent–offspring relationships, and their assignment to different gene pools.

Country	Number of genotypes	Number of varieties with relationships (%)	Number of relatives (%)	Number of genotypes assigned to each gene pool (%)			
				West	Center	East	Mosaic
France^1^	92	61 (66.3)	193	1 (1.6)	7 (11.5)	9 (14.8)	44 (72.1)
Morocco^2^	12	1 (8.3)	1 (0.5)	1 (100.0)			
Portugal^2^	12	2 (16.7)	2 (1.0)	2 (100.0)			
Spain^2^	75	18 (24.0)	21 (10.9)	15 (83.3)			3 (16.7)
Algeria^2^	24	3 (12.5)	3 (1.6)				3 (100.0)
France^2^	92	51 (55.4)	101 (52.3)	1 (2.0)	7 (13.7)	4 (7.8)	39 (76.5)
Tunisia^2^	11	1 (9.1)	1 (0.5)	1 (100.0)			
Italy^2^	92	28 (30.4)	42 (21.8)		12 (42.9)	3 (10.7)	13 (46.4)
Croatia^2^	8	3 (37.5)	5 (2.6)		1 (33.3)		2 (66.7)
Slovenia^2^	5	2 (40.0)	2 (1.0)		1 (50.0)		1 (50.0)
Greece^2^	12	1 (8.3)	1 (0.5)				1 (100.0)
Egypte^2^	17						
Cyprus^2^	2						
Lebanon^2^	4						
Syria^2^	37	5 (13.5)	14 (7.3)	1 (20.0)	1 (20.0)	2 (40.0)	1 (20.0)
**Total** **^2^**	**395** **^3^**	**115 (29.1)**	**193 (100.0)**	**21 (18.3)**	**22 (19.1)**	**9 (7.8)**	**63 (54.8)**

^1^Varieties identified as putative offspring.

^2^Varieties identified as putative parents.

^3^Eight genotypes were similar or genetically close between FOGB and WOGBM collections.

**Figure 10:**
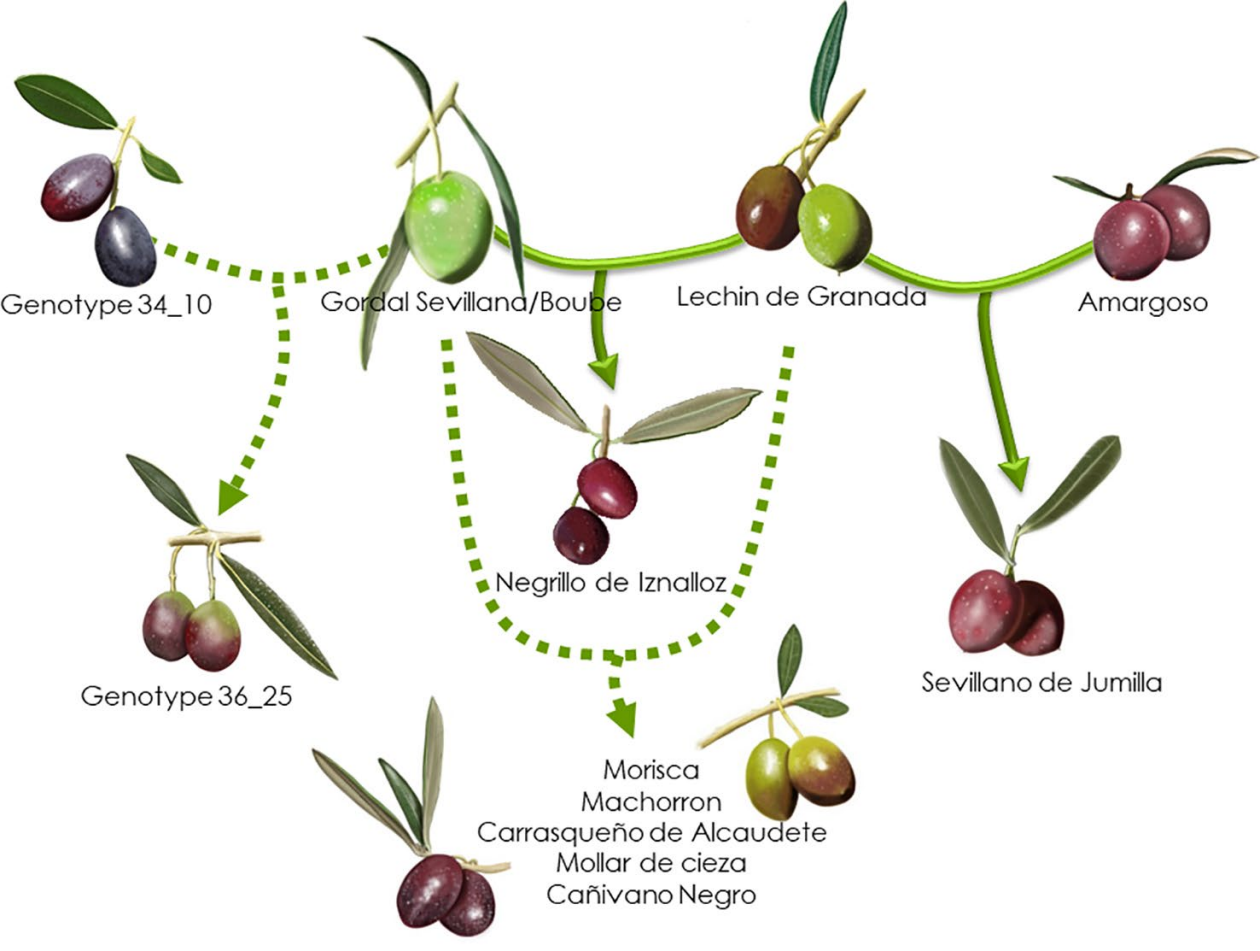
Parentage relationships of French varieties based on the likelihood approach. The parentage relationships are illustrated with the highest LOD_pp_ value and no allele mismatch (full line) or with an allele mismatch at one locus (dashed line).

Based on the 193 putative parent–offspring pairs, parentage relationships were examined by searching parental pairs with the likelihood approach. All varieties having at least one putative parent–offspring, as validated by at least one approach among the three used for a single parent search, were analyzed, including 86 French genotypes and 155 other Mediterranean varieties (**Supplementary Table S5**). A threshold LOD at 13.7 allowed us to define the success rate at 99.7% in detecting the most likely parental pair of the offspring based on the highest LOD score (**Figure 6B**). The French ‘Boube’ variety and the Spanish ‘Lechin de Granada’ variety were identified as the most likely parental pair for six Spanish varieties (**Table 5**; **Figure 10**): ‘Negrillo de Iznalloz’ was assigned with the highest LOD_pp_ value (23.6) and no allele mismatch, while the remaining most likely offspring were assigned at a LODpp ranging from 13.81 to 15.76, with an allele mismatch at one locus (**Table 5**). Moreover, the ‘Boube’ variety was identified as one of the most likely parents of the French ‘36_25’ genotype, with a LODpp at 15.69 and one mismatch at locus DCA16 (**Table 5**), while the ‘Lechin de Granada’ variety was identified as the most likely parent of the ‘Sevillano de Jumilla’ variety, with no allele mismatch. The ‘Boube’ variety harbors the E1-2 maternal haplotype, and thus could not be the mother of the seven identified offspring that shared the E1-1 maternal haplotype. Surprisingly, we detected only one pair of parents involving the ‘Cailletier’ variety (**Table 5**), despite the high number of putative parent–offspring pairs detected using the single-parent search (24; **Supplementary Table S4**).

**Table 5:** The most likely parental pairs of 10 varieties including Cailletier and two French genotypes based on the highest LODpp.

Cultivar name	N° Accession	Country	Assignation Q>0.8	Maternal lineage	LODpp	Incompatible markers	Putative pair parents	N° Accession	Country	Assignation Q > 0.8	Maternal lineage
Negrillo de Iznalloz	352	Spain	Western	E 1-1	23.6		Lechin de Granada	340	Spain	Western	E 1-1
							Boube	36_11	France	Western	E 1-2
Morisca	245	Spain	Western	E 1-1	15.756	DCA18	Lechin de Granada	340	Spain	Western	E 1-1
							Boube	36_11	France	Western	E 1-2
Machorron	247	Spain	Western	E 1-1	15.43	DCA9	Lechin de Granada	340	Spain	Western	E 1-1
							Boube	36_11	France	Western	E 1-2
Carrasqueño de Alcaudete	225	Spain	Western	E 1-1	14.495	DCA16	Lechin de Granada	340	Spain	Western	E 1-1
							Boube	36_11	France	Western	E 1-2
Mollar de cieza	348	Spain	Western	E 1-1	14.222	DCA18	Lechin de Granada	340	Spain	Western	E 1-1
							Boube	36_11	France	Western	E 1-2
Cañivano Negro	224	Spain	Western	E 1-1	13.812	DCA18	Lechin de Granada	340	Spain	Western	E 1-1
							Boube	36_11	France	Western	E 1-2
36_25	36_25	France	Mosaic	E 1-1	15.695	DCA16	34_10	34_10	France	Mosaic	E 1-1
							Boube	36_11	France	Western	E 1-2
Sevillano de Jumilla	272	Spain	Western	E 1-1	17.316		Lechin de Granada	340	Spain	Western	E 1-1
							Amargoso	219	Spain	Mosaic	E 1-1
9_01	9_01	France	Mosaic	E 1-1	20.634	GAPU103	Verdanel	10_1	France	Mosaic	E 1-1
							37_02	37_02	France	Mosaic	E 1-1
Cailletier	32_33	France	Central	E 1-1	28.831	DCA9	Cima di Melfi	92	Italy	Central	E 1-1
							Karme	640	Syria	Central	E 1-1

Maternal lineage, inferred ancestry (Q) among clusters at K = 3 for each genotype and LODpp values are indicated.

### Sampling Varieties to Represent French Olive Genetic Diversity

A core collection was defined according to the two-step method proposed by El Bakkali et al. (2013b) (**Supplementary Figure S3**). Forty-three genotypes (46.7%) were necessary to capture the 191 alleles in the FOGB collection. Based on half of the initial sample size of 43 (23.9%), a primary core collection of 22 genotypes was constructed (CC_22_; **Supplementary Table S7**). The 22 entries thus allowed the capture of 169 alleles (88.5%), three maternal haplotypes (18 E1-1, 1 E2-1, and 3 E3-1; 50%), and 17 reference varieties (**Supplementary Table S8**). This primary core collection (CC_22_) was used as a kernel with Mstrat to capture the remaining alleles. Hence, 43 entries (CC_43_; 46.7%) were sufficient to capture the total diversity. 50 sets of 43 French varieties were generated using Mstrat as the CC_43_ (**Supplementary Table S7**).

No differences were observed in the expected heterozygosity (*He*; Nei, 1987) in 50 independent runs. In addition to the 22 varieties used as a kernel, 18 varieties were found to be common in all of the 50 independent runs, while a combination of three complement genotypes could be selected among a panel of seven genotypes to capture the total number of alleles (**Supplementary Table S7**). Among the 25 genotypes captured, 15 were validated as reference French varieties.

The French core collection CC_43_ was arbitrarily selected (**Supplementary Table S8**). Among the 43 entries sampled, only four *cpDNA* haplotypes were captured: E1.1 (34 individuals), E1.2 (1), E2.1 (4), and E3.1 (4; **Supplementary Table S8**). Moreover, 31 reference French varieties were selected among the 43 entries of CC_43_. To select all reference varieties, the size was increased to 75 cultivars, which represented the third level of the French core collection (CC_75_; **Supplementary Table S8**). Most of the varieties sampled in CC_43_ showed high admixture since 33 varieties (76.7%) belonged to more than one gene pool, while only six and four genotypes were assigned (with membership probabilities of Q ≥ 0.80) to central and eastern gene pools, respectively (**Supplementary Table S8**). Genotypes selected for the primary core collection (CC_22_) and for the core collection capturing all alleles (CC_43_) had the lowest frequency of parentage relationships (8% and 22%, respectively; **Supplementary Table S8**). This pattern is in line with the findings obtained with the approach used to construct the core collection favoring genotypes without genetic relatedness.

## Discussion

Our study first allowed us to generate a database for efficient identification of French varieties. We took advantage of this genetic characterization to investigate French olive genetic diversity and assess the importance of local genetic resources and their associated agroecosystems in the cultivated olive tree diversification process.

### Diversification of French Olive Germplasm by Admixture

Primary selection and secondary diversification are two key processes in the history of olive domestication (Khadari and El Bakkali, 2018). Diversification can be viewed as a process that is driven mainly by farmer selection of trees harboring interesting traits. As this selection occurs within the agroecosystem, selected trees are most likely derived from crosses between varieties or previously selected clones, sometimes with pollen coming from feral or wild olive trees (for review, see Gaut et al., 2015; Besnard et al., 2018). Sociohistorical and ethnobiological investigations of traditional olive agroecosystems in northern Morocco have highlighted strong links between selected trees from clonally and seed propagated trees, indicating the continuing roles of cultivated, feral, and wild olive trees in the diversification process (Aumeeruddy-Thomas et al., 2017). Here we also showed an admixed origin of French varieties, suggesting a diversification process involving local and introduced genetic resources. Among the 92 French genotypes, 68 (73.9%) were admixed as they were assigned to more than one group with Q < 0.80. Most of them (82.6%) harbored the eastern maternal lineage [i.e. haplotypes E1-1 (73) and E1-2 (3)] originating from the eastern Mediterranean Basin, and it was introduced in the westernmost regions *via* the diffusion of oleiculture (Besnard et al., 2013b).

As previously suggested by several authors (Besnard et al., 2001a; Baldoni et al., 2006; Belaj et al., 2007; Breton et al., 2008; Besnard et al., 2013a; Diez et al., 2015), in our following arguments we assumed that the French olive germplasm was mainly derived from a diversification process involving local genetic resources, in addition to the introduction of cultivated olives belonging mainly to the Q2 genepool (central Mediterranean), as well as the Q1 genepool (western Mediterranean). First, we observed a clear genetic pattern derived from admixture germplasm from the central Mediterranean area, including French local genetic resources, as previously reported by Haouane et al. (2011); Diez et al. (2012), and El Bakkali et al. (2013a). Second, these local genetic resources harbored a maternal lineage from the eastern primary domestication center (Besnard et al., 2013b). Third, despite the reduction in allelic diversity (22.4%) as compared to Mediterranean cultivated olive, the French germplasm showed a similar expected heterozygosity and pairwise genetic distance pattern compared to Mediterranean olive germplasm, indicating that admixture was likely a consequence of this pattern. Fourth, we highlighted that approximately half of the *ex situ* collection of Porquerolles (46.7%) was necessary to capture all of the French diversity, which was mainly classified in the mosaic Mediterranean group. Finally, we observed substantial parentage relationships (parent–offspring) at a local scale within French varieties and at a regional scale between French, Italian, or Spanish varieties, indicating that selection from crossing between varieties was likely a key varietal diversification process within French agroecosystems and neighboring regions.

### French Agroecosystems as a Bridge Between Italy and Spain for Olive Diversification

Identical and nearly identical genotypes were identified among French and other Mediterranean germplasm. This is evidence in favor of the translocation of varieties between distant regions (e.g. Besnard et al., 2001b; Haouane et al., 2011; Trujillo et al., 2014). Interestingly, we report for the first time the high genetic similarity between ‘Cailletier’, a major French variety, and the Italian ‘Frantoio’ variety. These two genotypes were here distinguished by only one allele on the reference genotype of each variety and may have represented distinct clones propagated from a single genotype (e.g. due to clonal selection; Bellini et al., 2008). A similar pattern was noted for the French ‘Petit Ribier’ variety and the Italian ‘Moraiolo’, variety as previously observed by Pinatel (2015) in a study using morphological descriptors. Both ‘Cailletier’ and ‘Petit Ribier’ varieties are mainly cultivated in southeastern France (Var, Alpes de Haute Provence and Alpes Maritimes), while ‘Frantoio’ and ‘Moraiolo’ are notably cultivated in Tuscany (Italy). These two varieties are also known to have been introduced in Corsica from Italy under the polyclonal denomination ‘Ghermana’ (Besnard et al., 2001b; Bronzini de Caraffa et al., 2002). Otherwise, the local French ‘Boube’ variety was found to be genetically similar to that of the oldest Spanish variety, i.e. ‘Gordal Sevillana’, and it was the only French variety clearly assigned to the western genepool (Q1). Pinatel (2015) considered ‘Boube’ as a local variety (only present in three distant orchards in Alpes de Haute Provence, with a single centennial tree per orchard) that was probably cultivated in southern France in ancient times. Here our results suggest that the variety was probably introduced from the Iberian Peninsula due to the large olive fruit size. Its past importance in the western Mediterranean Basin needs to be reviewed as it was spread over broad areas (at least from Andalusia to southeastern France) and then was involved in varietal diversification, particularly in Spain, but also elsewhere (Diez et al., 2015).

Beyond the substantial parentage relationships within the French germplasm (53.89%), we clearly identified relationships (parent–offspring) between French and Italian varieties, as well as French and Spanish varieties, mainly based on ‘Cailletier’/’Frantoio’ and ‘Boube’/’Gordal Sevillana’ varieties, since they harbored the highest number of putative parent–offspring pairs (24 and 36, respectively). Interestingly, the ‘Cailletier’/’Frantoio’ variety assigned to the central cluster had robust relationships (parent–offspring) with varieties from Italy which belonged to the same cluster, as well as the ‘Petit Ribier’/’Morailo’ variety, while the ‘Boube’ variety displayed parentage relationships from Spanish germplasm. We observed that ‘Cailletier’/’Frantoio’ and ‘Petit Ribier’/’Morailo’ were the main progenitors of Italian/French varieties, while ‘Boube’/’Gordal Sevillana’ was the main progenitor of Spanish/French varieties. As previously reported by Diez et al. (2015), we confirmed that ‘Gordal Sevillana’ was one of the main progenitors of Spanish germplasm, but strikingly we found that it was likely the male parent of six French varieties, i.e. ‘Clermontaise’ and ‘Courbeil’, which are cultivated in southwestern area (Hérault and Pyrénées Orientales; Moutier et al., 2004) bordering northeastern Spain (Catalonia). This Spanish variety was considered by Diez et al. (2015) as being one of the main founders of the western genepool (Q1), and based on our results we hypothesize that it was also the founder of part of the French germplasm assigned to the Mosaic genetic group. In addition, we noted for the first time that ‘Frantoio’ and ‘Morailo’ were putative progenitors of numerous Italian and French varieties. This result also suggests that these two varieties have been major progenitors within the Central Mediterranean group (Q2).

### French Olive Agroecosystems as Varietal Diversification Incubators

Surprisingly, despite the limited French olive growing area (southern continental France and Corsica), we identified a high number of varieties in the *ex situ* collection of Porquerolles, including a panel of at least 30 currently cultivated varieties (Moutier et al., 2004; Moutier et al., 2011). French olive growing is still mainly founded on a traditional system involving a diverse range of crops and varieties (Pinatel, 2015). This could be viewed as a key factor favoring varietal diversity, as previously noted by several authors (Gemas et al., 2004; Khadari et al., 2008; Kaya et al., 2013; Marra et al., 2013; Las Casas et al., 2014; Xanthopoulou et al., 2014).

Here, we assumed that the French varietal diversity could mainly be explained by active farmer selection, probably due to the impact of relatively frequent climatic accidents on local germplasm. Indeed, French olives are cultivated along the northern rim of the olive growing area where frost events are frequent—the last major one, in 1956, caused substantial damage in olive orchards, thus negatively impacting the socioeconomic sector (Pinatel, 2015). Moreover, we report for the first time that French genetic resources displayed substantial parentage relationships involving both local and foreign varieties (55 and 60, respectively), with more than half of the parent–offspring pairs occurring in local French germplasm (53.89%; **Table 1**). A similar pattern was observed in the western group (Q1 cluster), where the average number of first-degree relationships (full siblings or parent–offspring) was 16.25, while it was 2.0 in the Q2 (central Mediterranean) and Q3 (eastern Mediterranean) genetic clusters (Diez et al., 2015). Similarly, in grapevine, selection *via* crossing was previously identified by investigating the parentage relationships of ‘Chardonnay’, ‘Gamay’, and other wine grapes grown in northeastern France (Bowers et al., 1999). In their extended parentage analysis of the INRA grape germplasm repository (France; 2,344 unique genotypes), Lacombe et al. (2013) identified the full parentage of 828 cultivars including 447 traditional cultivars, which are likely derived from farmer selection in traditional agroecosystems. These processes have been reported in other perennial fruit cropping systems such as apricot, which is seed-propagated in oasis agroecosystems in the southern Maghreb region (Bourguiba et al., 2012; Bourguiba et al., 2013). Since the second half of 20th century, so-called “modern” perennial fruit cropping systems were managed with a single variety using agronomic practices that fostered yield improvement. They gradually replaced traditional agroecosystems which were based on higher diversity of crops and varieties than modern systems. Such diversified agroecosystems may still be found in mountainous areas around the Mediterranean Basin, as described, for instance, by Aumeeruddy-Thomas et al. (2017) in North Morocco. In southern France where olive and grapevine are often cultivated in the same locations, the olive varieties identified in the present study were mainly derived from crosses between local and foreign genetic resources, as we revealed by the parentage analysis.

## Conclusion

Our results provide a clear picture regarding the importance of farmer selection in the olive varietal diversification process in traditional French agroecosystems. Indeed, we observed substantial parentage relationships within French olive germplasm and the proportion of parent–offspring pairs was still high (45.08% out the 193 putative parent–offspring pairs), even when not considering the Italian ‘Frantoio’ variety or the Spanish ‘Gordal Sevillana’ variety. Otherwise, we observed a pattern of parentage relationships from crossing: (*i*) between French and Spanish varieties within agroecosystems in southwestern France, especially in the Pyrénées Orientales area, and (*ii*) between French and Italian varieties in the southeastern France, particularly in the Alpes Maritimes area. We thus argue in favor of active farmer selection founded mainly on local French varieties, probably due to frequent climatic accidents such as frost. When examining diversification processes at the regional scale in all southern European countries, we consider that French agroecosystems are incubators for olive diversification and serve as a bridge between Italy and Spain (Khadari et al., 2003), thus highlighting the importance of diversification as one of the two key processes in the history of olive domestication (Khadari and El Bakkali, 2018).

## Data Availability Statement

All datasets for this study are included in the article/**Supplementary Material**.

## Author Contributions

BK designed the research and wrote the manuscript with AEB and GB. AEB, LE, and CT performed microsatellite genotyping. BK and CP checked the reference list of French varieties. BK and AEB performed the data analysis. BK, AEB, and GB interpreted the data analysis. All co-authors participated in approving the final manuscript.

## Funding

This work was conducted at the AGAP research unit and supported by the OliveMed/Agropolis Fondation N° 1202-066 project through the Investissements d’avenir/Labex Agro ANR-10-Labex-0001-01 managed by the French National Research Agency (ANR). GB was also supported by the LABEX TULIP (ANR-10-LABX-0041) and CEBA (ANR-10-LABX-25-01).

## Conflict of Interest

The authors declare that the research was conducted in the absence of any commercial or financial relationships that could be construed as a potential conflict of interest.

## Supplementary Material

Table S1 List of accessions from the WOGB and FOGB collections (529 accessions); 416 and 113, respectively. Accessions are classified according to origin, maternal lineage, inferred ancestry (Q) among clusters at *K* = 3, and SSR code (code for our genotyping analyses). French cultivars showing relationships with other cultivars are indicated.

Table S2 List of French varieties with genetically close SSR profiles which are distinct by one or two dissimilar alleles and considered as somatic mutations

Table S3 Optimal loci combination to discriminate the 92 French genotypes identified among the 113 accessions

Table S4 Comparison of allelic richness between the FOGB and WOGB collections. *Na* = Number of alleles; *Ar* = allelic richness.

Table S5 List of French genotypes showing putative parent relationships with other genotypes using three approaches; the exclusion (mismatch marker), the log-likelihood ratio (LODp) and the exclusion-Bayes’ theorem. Maternal lineage and inferred ancestry (Q) among clusters at *K* = 3 are indicated for each genotype, as well as results for each parentage approach.

Table S6 Number and proportion of genotypes from different countries assigned to each of the three gene pools identified by under the assignation probability of Q ≥ 0.8.

Table S7s List of different nested French core collections with different sample sizes (_22_, CC_43_, and CC_75_). (x) Corresponds to the presence of the accession in each core collection. Twenty-two varieties (CC_22_) were sampled by the CoreHunter program when optimizing the Shannon and Weaver index “*Sh strategy*”. CC_22_ was then used as a kernel in the Mstrat program to reconstruct the extended core collections: 43 varieties (CC_43_) allowed us to capture all of the observed alleles, and 75 varieties (CC_75_) included the remaining reference varieties not sampled in CC_43_.

Table S8 Description of different nested core collections constructed from the FOGB collection.

Figure S1 Optimal number of clusters using the program and inferred population structure from *K* = 2 to *K* = 4 for 395 distinct genotypes from both collections. *H′* represents the similarity coefficient between runs for each *K*, and Δ*K* represents the *ad hoc* measure of Evanno et al. (2005).

Figure S2 A network of French and Mediterranean varieties showing parentage relationships according to the genetic structure of varieties. Names of varieties and their assignment to different gene pools are indicated.

Figure S3 Sampling efficiency based on the ability to capture the genetic diversity *via* the M-strategy (M-method) compared to a random strategy.
