# Supplementary material for: Cultivated Olive Diversification at Local and Regional Scales: Evidence From the Genetic Characterization of French Genetic Resources
Source: Front Plant Sci. 2019 Dec 24;10:1593. doi: 10.3389/fpls.2019.01593 (PMC6937215; doi:10.3389/fpls.2019.01593)
Supplement: Table S3 — Optimal loci combination to discriminate the 92 French genotypes identified among the 113 accessions [file Table_3.docx]

**Table S3.** Optimal loci combination to discriminate the 92 French genotypes identified among the 113 accessions.

| **No. of loci** | **Loci combination** | **Number of genotypes** | **Proportion (%)** |
| --- | --- | --- | --- |
| 1 | DCA09 | 39 | 42.4 |
| 2 | DCA09 + DCA04 | 82 | 89.1 |
| 3 | DCA09 + DCA04 + GAPU101 | 92 | 100 |
