# Supplementary material for: Cultivated Olive Diversification at Local and Regional Scales: Evidence From the Genetic Characterization of French Genetic Resources
Source: Front Plant Sci. 2019 Dec 24;10:1593. doi: 10.3389/fpls.2019.01593 (PMC6937215; doi:10.3389/fpls.2019.01593)
Supplement: Table S4 — Comparison of allelic richness between the FOGB and WOGB collections. Na = Number of alleles; Ar = allelic richness. [file Table_4.docx]

**Table S4.** Comparison of the allelic richness between FOGB and WOGBM collections. Number of alleles (*Na*), allelic richness (*Ar*)

| **N** | **Locus** | ***Na^1^*** | ***Na^2^*** | ***Ar^3^*** | ***Ar^4^*** |  |
| --- | --- | --- | --- | --- | --- | --- |
| **1** | DCA01 | 7 | 19 | 5.85 | 8.85 |  |
| **2** | DCA03 | 7 | 14 | 6.99 | 9.49 |  |
| **3** | DCA04 | 20 | 34 | 15.95 | 17.73 |  |
| **4** | DCA05 | 11 | 12 | 9.68 | 9.56 |  |
| **5** | DCA08 | 15 | 21 | 11.54 | 13.01 |  |
| **6** | DCA09 | 18 | 24 | 14.00 | 16.55 |  |
| **7** | DCA11 | 11 | 24 | 8.38 | 14.23 |  |
| **8** | DCA14 | 9 | 15 | 8.10 | 10.04 |  |
| **9** | DCA15 | 4 | 7 | 3.88 | 5.20 |  |
| **10** | DCA16 | 10 | 35 | 8.75 | 16.27 |  |
| **11** | DCA18 | 10 | 17 | 9.21 | 12.00 |  |
| **12** | GAPU59 | 7 | 11 | 5.99 | 6.93 |  |
| **13** | GAPU71A | 5 | 16 | 4.38 | 6.96 |  |
| **14** | GAPU71B | 6 | 8 | 5.50 | 6.84 |  |
| **15** | GAPU101 | 8 | 13 | 7.88 | 9.63 |  |
| **16** | GAPU103A | 14 | 26 | 12.28 | 14.90 |  |
| **17** | EMO03 | 11 | 13 | 8.72 | 10.40 |  |
| **18** | EMO90 | 5 | 9 | 5.00 | 6.88 |  |
| **19** | UDO-017 | 6 | 9 | 5.94 | 7.24 |  |
| **20** | UDO-036 | 7 | 12 | 5.75 | 8.26 |  |
|  | **Mean** | **9.55** | **16.95** | **8.19** | **10.54** |  |
|  | **Total** | **191** | **339** | **163.76** | **210.96** |  |

^1^Number of alleles detected in FOGB (92 genotypes)

^2^Number of alleles detected in the WOGBM (311 genotypes)

^3^Allelic richness standardised at value G = 92 individuals for the FOGB collection

^4^Allelic richness standardised at value G = 92 individuals for the WOGBM collection

The pairwise value Ar^3^/ Ar^4^ is significantly different (Kruskal-Wallis test; P-value=0.032)
