# Supplementary material for: Cultivated Olive Diversification at Local and Regional Scales: Evidence From the Genetic Characterization of French Genetic Resources
Source: Front Plant Sci. 2019 Dec 24;10:1593. doi: 10.3389/fpls.2019.01593 (PMC6937215; doi:10.3389/fpls.2019.01593)
Supplement: Table S6 — Number and proportion of genotypes from different countries assigned to each of the three gene pools identified by under the assignation probability of Q ≥ 0.8. [file Table_6.docx]

**Table S6.** Number and proportion of genotypes from different countries assigned to each of the three gene pools identified by Structure program under the assignation probability of Q ≥ 0.8.

|  | **Country** | **Number of genotypes** | **Total of genotypes assigned by**  **Structure (%)** | **Number of genotypes assigned to each gene pool (%)** | | | |
| --- | --- | --- | --- | --- | --- | --- | --- |
|  |  |  |  | **West Medit.** | **Center Medit.** | **East Medit.** | **Mosaic** |
| 1 | Morocco | 12 | 7 (58.3) | 5 (41.7) | 2 (16.7) |  | 5 (41.7) |
| 2 | Portugal | 12 | 8 (66.7) | 8 (66.7) |  |  | 4 (33.3) |
| 3 | Spain | 75 | 56 (74.7) | 51 (68.0) | 5 (6.7) |  | 19 (25.3) |
| **Western Mediterranean** | | **99** | **71 (71.7)** | **64 (64.6)** | **7 (7.1)** |  | **28 (28.3)** |
| 4 | Algeria | 24 | 9 (37.5) |  | 8 (33.3) | 1 (4.2) | 15 (62.5) |
| 5 | Tunisia | 11 | 5 (45.5) | 1 (9.1) | 2 (18.2) | 2 (18.2) | 6 (54.5) |
| 6 | France | 92 | 24 (26.1) | 1 (1.1) | 8 (8.7) | 15 (16.3) | 68 (73.9) |
| 7 | Italy | 92 | 46 (50.0) | 3 (3.3) | 32 (34.8) | 11 (12.0) | 46 (50.0) |
| 8 | Croatia | 8 | 2 (25.0) |  | 1 (12.5) | 1 (12.5) | 6 (75.0) |
| 9 | Slovenia | 5 | 1 (20.0) |  | 1 (20.0) |  | 4 (80.0) |
| 10 | Greece | 12 | 5 (41.7) |  | 2 (16.7) | 3 (25.0) | 7 (58.3) |
| **Central Mediterranean** | | **244** | **92 (37.7)** | **5 (2.0)** | **54 (22.1)** | **33 (13.5)** | **152 (62.3)** |
| 11 | Egypte | 17 | 10 (58.8) |  |  | 10 (58.8) | 7 (41.2) |
| 12 | Cyprus | 2 | 2 (100.0) |  |  | 2 (100.0) |  |
| 13 | Lebanon | 4 | 3 (75.0) |  |  | 3 (75.0) | 1 (25.0) |
| 14 | Syria | 37 | 32 (86.5) | 1 (2.7) | 2 (5.4) | 29 (78.4) | 5 (13.5) |
| **Eastern Mediterranean** | | **60** | **47 (78.3)** | **1 (1.7)** | **2 (3.3)** | **44 (73.3)** | **13 (21.7)** |
| **Total** | | **395^1^** | **210 (52.1)** | **70 (17.4)** | **63 (15.6)** | **77 (19.1)** | **193 (47.9)** |

^1^ Eight genotypes were similar or genetically close between FOGB and WOGBM collections.
