# Supplementary material for: Cultivated Olive Diversification at Local and Regional Scales: Evidence From the Genetic Characterization of French Genetic Resources
Source: Front Plant Sci. 2019 Dec 24;10:1593. doi: 10.3389/fpls.2019.01593 (PMC6937215; doi:10.3389/fpls.2019.01593)
Supplement: Table S8 — Description of different nested core collections constructed from the FOGB collection. [file Table_8.docx]

**Table S8.** Description of different nested core collections constructed from FOGB collection.

|  |  | **FOGB** | **CC_22_** | **CC_43_** | **CC_75_** |
| --- | --- | --- | --- | --- | --- |
| Sample size |  | 92 | 22 | 43 | 75 |
| Number of reference varieties |  | 63 | 17 | 31 | 63 |
| Number of alleles |  | 191 | 169 | 191 | 191 |
| Number of cpDNA haplotypes | E1.1 | 73 | 18 | 34 | 58 |
|  | E1.2 | 1 |  |  | 1 |
|  | E1.4 | 2 |  | 1 | 2 |
|  | E2.1 | 8 | 1 | 4 | 8 |
|  | E3.1 | 7 | 3 | 4 | 6 |
|  | E3.3 | 1 |  |  |  |
| Assignation of different gene pools (Structure program ≥ 0.8) | Western | 1 |  |  | 1 |
|  | Central | 8 | 4 | 6 | 8 |
|  | Eastern | 15 |  | 4 | 13 |
|  | Mosaic | 68 | 18 | 33 | 53 |
| Number of putative parent-offspring pairs |  | 193 | 15  (8%) | 43  (22%) | 159  (82%) |
| Number of putative parent-offspring pairs within French germplasm |  | 104 | 11 (11%) | 32  (31%) | 78  (75%) |
| Number of putative parent-offspring pairs between French and Mediterranean varieties |  | 89 | 4  (4%) | 11  (12%) | 81  (91%) |
