# Supplementary figures and images for: Cultivated Olive Diversification at Local and Regional Scales: Evidence From the Genetic Characterization of French Genetic Resources
Source: Front Plant Sci. 2019 Dec 24;10:1593. doi: 10.3389/fpls.2019.01593 (PMC6937215; doi:10.3389/fpls.2019.01593)

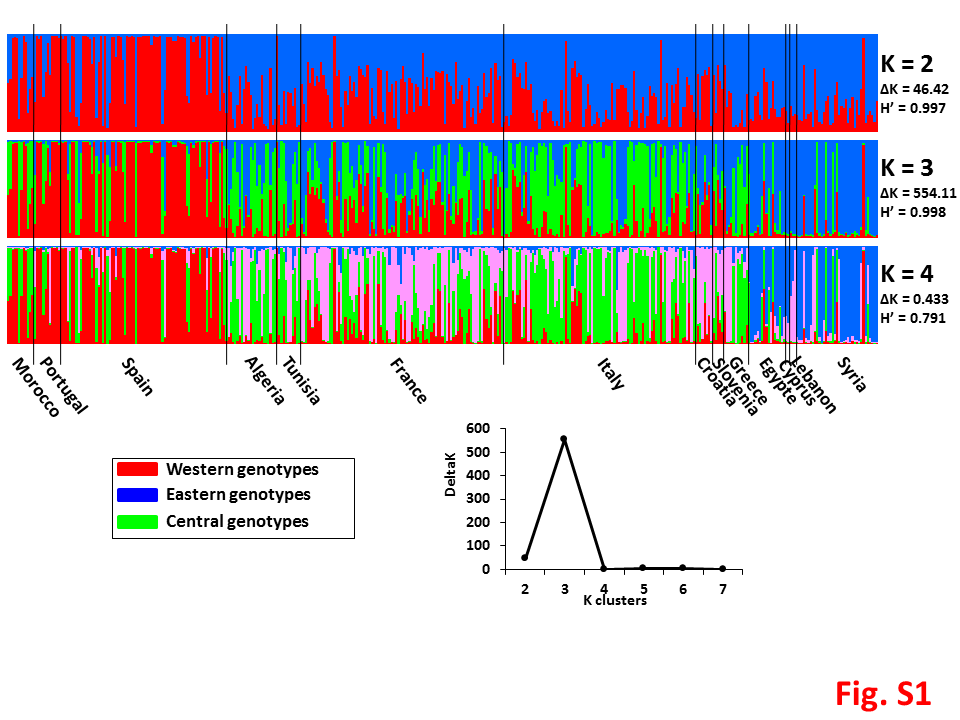

Supplement: Figure S1 — Optimal number of clusters using the program and inferred population structure from K = 2 to K = 4 for 395 distinct genotypes from both collections. H′ represents the similarity coefficient between runs for each K, and ΔK represents the ad hoc measure of Evanno et al. (2005). [file Image_1.tif]

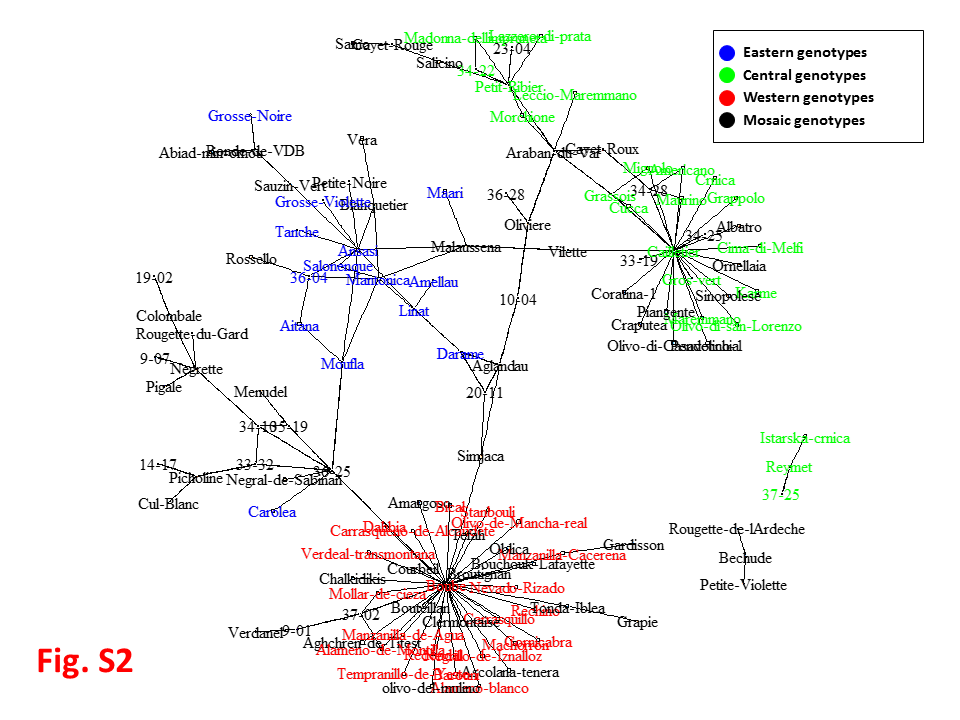

Supplement: Figure S2 — A network of French and Mediterranean varieties showing parentage relationships according to the genetic structure of varieties. Names of varieties and their assignment to different gene pools are indicated. [file Image_2.tif]

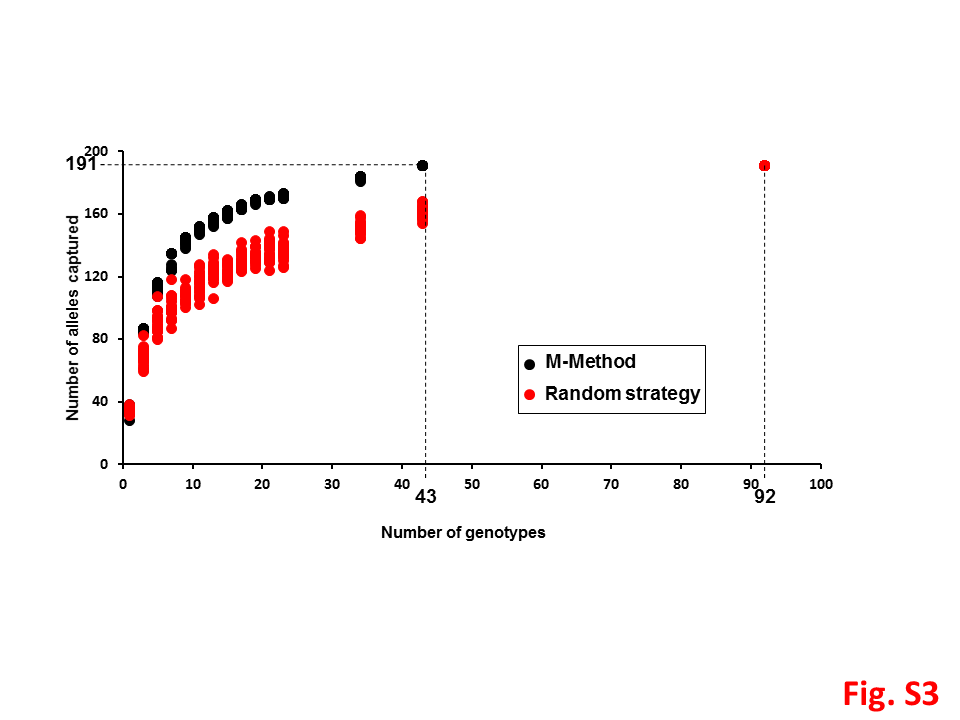

Supplement: Figure S3 — Sampling efficiency based on the ability to capture the genetic diversity via the M-strategy (M-method) compared to a random strategy. [file Image_3.tif]
